# The Possible Crystallization Process in the Origin of Bacteria, Archaea, Viruses, and Mobile Elements

**DOI:** 10.3390/biology14010003

**Published:** 2024-12-24

**Authors:** Akari Yoshimura, Masayuki Seki

**Affiliations:** Division of Biochemistry, Faculty of Pharmaceutical Sciences, Tohoku Medical and Pharmaceutical University, Sendai 981-8558, Japan; akari-yo@tohoku-mpu.ac.jp

**Keywords:** the last universal common ancestor, submarine alkaline hydrothermal vent, TBP, σ-factor, DNA polymerase, recombinase, error catastrophe

## Abstract

We propose a hypothesis for the simultaneous emergence of bacteria, archaea, viruses, and mobile elements by sequential and concrete biochemical pathways. The emergence process can be considered analogous to crystallization, where genetic and biochemical systems stabilize as organisms evolve from their common ancestor, the LUCA, which was a non-free-living pool of single operon type genomes including double-stranded (ds) DNA at an ancient submarine alkaline vent. Each dsDNA operon was transcribed by different systems in σ, TFIIB, or TBP genomes. Double-stranded DNA operons can fuse and stabilize through the action of specific transcription systems, leading to differentiation between the Bacteria (σ genome) and Archaea (TBP genome) domains. Error catastrophe can be overcome by the parallel gain of DNA replication and DNA repair mechanisms in both genomes. Enlarged DNA enabled efficient local biochemical reactions. Both genomes independently recruited lipids to facilitate reactions by forming coacervates at the chamber of the vent. Bilayer lipid membrane formation, proto-cell formation with a permeable membrane, proto-cell division, and the evolution of membrane-associated biochemistry are presented in detail. Simultaneous crystallization of systems in non-free-living bacteria and non-free-living archaea triggered the co-crystallization of primitive viruses and mobile elements. An arms race between non-free-living cells and primitive viruses finally led to free-living cells with a cell wall and mature viruses.

## Simple Summary

How free-living cells and mature viruses arose on ancient Earth remains elusive. In general, crystallization is the natural or artificial process by which solid crystals form from a homogeneous solution. In this study, the process by which cells and viruses spontaneously form from solutions containing various biomolecules is metaphorically referred to as crystallization. We propose that the last universal common ancestor (LUCA) was a non-free-living pool of single operon-type genomes, including double-stranded (ds) DNA, at an ancient submarine alkaline vent. Here, we show how the dsDNA operons were crystallized as free-living cells with a cell wall and mature viruses.

## 1. Introduction

**Figure 1:**
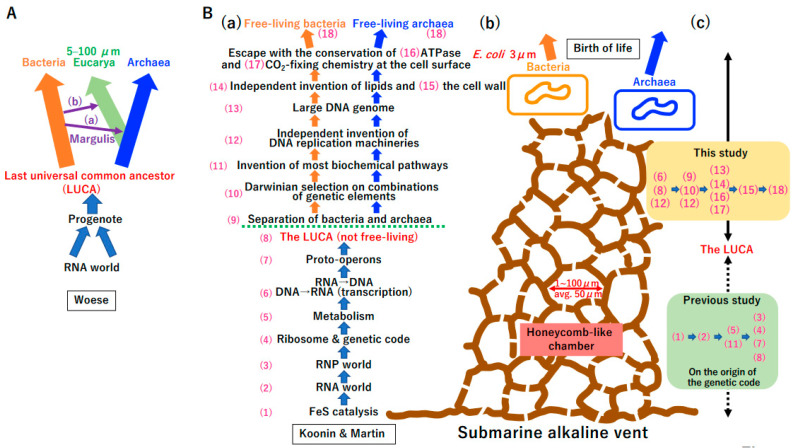
Crystallization of bacteria and archaea. (**A**) Woese’s model [5]. Phylogenetic analysis of rDNA revealed three domains of life, Bacteria, Archaea, and Eucarya [1]. (**a**) Symbiosis between Archaea and α-Proteobacteria could yield Eucarya (lower purple arrow). (**b**) Symbiosis between Eucarya and Cyanobacteria could yield Archaeplastida (upper purple arrow). The concepts in (**a**,**b**) were originally proposed by Margulis [3]. Eukaryotic cells range in size from 5 to 100 µm. Woese proposed that the Bacteria and Archaea domains arose from the crystallization of systems in the LUCA. Moreover, the LUCA might be derived from a progenote descended from an RNA world. (**B**) Submarine alkaline vent model. (**a**) The emergence of free-living bacteria and archaea at an ancient submarine alkaline vent was described as a stepwise process, (1)–(18) [24]. Koonin and Martin proposed that the LUCA was not a free-living cell. (**b**) Cartoon of a submarine alkaline vent. Extant vents as well as early vents have numerous honeycomb-like chambers with sizes of 1 to 100 µm (average 50 µm) [25]. *E. coli* (bacteria) is 3 µm long. (**c**) Steps proposed in the current study. We have previously proposed a hypothesis for the origin of the genetic code and evolution of the translation system [28]. The order of events described in (**B**(**a**)) differs from that described in previous study (green background) [28]. In this study, we proposed evolutionary steps from the LUCA to bacteria and archaea at an ancient submarine alkaline vent (yellow background).

A phylogenetic analysis of ribosomal DNA (rDNA) revealed three domains of life, Bacteria, Archaea, and Eucarya (Figure 1A) [1,2]. The theory that symbioses of Bacteria and Archaea yielded Eucarya (Figure 1A(a)) was proposed [3]. Subsequently, symbioses of Cyanobacteria and Eucarya could yield Archaeplastida (Figure 1A(b)) [3]. Woese proposed an order of cellular evolution from an RNA world [4] to the last universal common ancestor (LUCA) of Bacteria and Archaea mediated by a progenote (Figure 1A) [5,6].

The RNA world theory has been repeatedly denied, mainly due to the second law of thermodynamics [7,8,9,10], because synthesis of RNA and protein in the progenote, derived from the RNA world, should require materials and free energy in the manner of a ratcheting mechanism [11,12,13,14,15,16,17]. Thus, the first steps in evolutionary complexification proceeded through climbing a “staircase-ascending complexity ratchet”. A submarine alkaline hydrothermal vent (AHV) is an ideal birthplace of life, as originally proposed by Russell and Hall [18] (Supplementary Figure S1A). Under the second law of thermodynamics, an AHV could provide protons, electrons, and a variety of prebiotically synthesized organic molecules through green rust (the term “green rust” is used to describe a range of green crystalline chemical compounds that contain iron(II) and iron(III) cations, as well as the hydroxide anion and other anions such as carbonate, chloride, or sulfate) (Supplementary Figure S1B) [19,20]. Moreover, reactors mimicking an AHV produce ribose, deoxyribose, and peptides in vitro (Supplementary Figure S1B) [21,22]. Thus, the progenote might have somehow survived in an AHV under the second law of thermodynamics (Supplementary Figure S2). Moreover, the LUCA likely depended on a controlled environment (such as hydrothermal vents) to sustain its vital functions.

Based on the foundation of the original AHV theory [18,23], Koonin and Martin hypothesized that early cells evolved via 18 steps (Figure 1B(a)) at ancient submarine AHVs (Figure 1B(b)) [24]. Of note, the size of honeycomb-like chambers at both extant and fossil vents (Figure 1B(b)) [25] is nearly equivalent to the size of eukaryotic cells (Figure 1A). The LUCA was predicted to be non-free-living [5,24] with the extant genetic code, translation system, and hundreds of proteins (Supplementary Figure S2) [26,27].

Previously, we proposed a hypothesis to explain the origin of the genetic code and translation system (Supplementary Figure S2) [28]. The postulated scenario from an RNA world to the LUCA (the progenote in Supplementary Figure S2) (Figure 1B(c) lower green panel) [28] is different from that proposed by Koonin and Martin [24] (Figure 1B(a)). The final step in the emergence of bacteria and archaea from the LUCA was called “crystallization”, referring to the stabilization of genetic systems (Figure 1B(b)) by Woese [5].

In this study, we theoretically examined crystallization processes from the LUCA to extant cells. We hypothesize that crystallization was mainly driven by two physical laws (the second law of thermodynamics [29,30] and Eigen’s error catastrophe [31]). Error catastrophe can be defined as the cumulative loss of genetic information in a lineage of organisms due to high mutation rates. The term is used to refer to the accumulation of mutations to the point of the organism or virus becoming inviable. Remarkably, the order of steps (Figure 1B(c) upper yellow panel) toward crystallization differs from that proposed by Koonin and Martin [24] (Figure 1B(a)). Briefly, in accordance with the theory [24], the emergence of double-stranded DNA (dsDNA) preceded that of the LUCA. Additionally, two distinct types of DNA replication machineries evolved prior to the emergence of DNA enlargement, while the evolution of ATPase occurred subsequent to the appearance of the cell wall. Furthermore, the selfish element is postulated to have existed from the outset. In contrast, the present study posits that the emergence of dsDNA corresponds to the birth of LUCA. Furthermore, it is proposed that DNA enlargement drives the evolution of two or more DNA replication machineries. The emergence of ATPase is posited to precede that of the cell wall. Finally, the selfish proto-cell and primitive viruses are posited to have emerged just before the emergence of free-living cells and mature viruses. Complete scenarios for crystallization processes are presented.

Briefly, the possible crystallization process in the origin of bacteria, archaea, viruses, and mobile elements is mainly divided into the following seven stages: (stage 1) from the progenote to the LUCA; (stage 2) the end of the LUCA; (stage 3) enlargement of the DNA genome associated with the “DNA replication divide” and overcoming Eigen’s error catastrophe; (stage 4) formation of coacervate, leading to proto-cells with permeable membranes; (stage 5) evolution of membrane proteins on permeable membranes and the beginning of “Omnis cellula a cellula”; (stage 6) the end of proto-cells with permeable membranes and the beginning of non-free-living cells with non-permeable membranes; and (stage 7) crystallization (emergence of free-living cells with cell walls and co-emergence of viruses and mobile elements).

## 2. Materials and Methods

Based on referenced materials, the current article was developed by outlining the logical sequence in which various biochemical pathways present in existing cells would have emerged as the earliest cells on Earth.

## 3. Results and Discussion

### 3.1. Stage 1 (From the Progenote to the LUCA)

The progenote might have consisted of RNA (Supplementary Figure S2). By contrast, a double-stranded DNA genome could have been invented in the LUCA. Stage 1 describes how the DNA strand (Section 3.1.1), transcription using a DNA template (Section 3.1.2), and replication of double-stranded DNA (Section 3.1.3) emerged during the transition from the progenote to the LUCA.

#### 3.1.1. Double-Stranded U-DNA on a Single Operon

**Figure 2:**
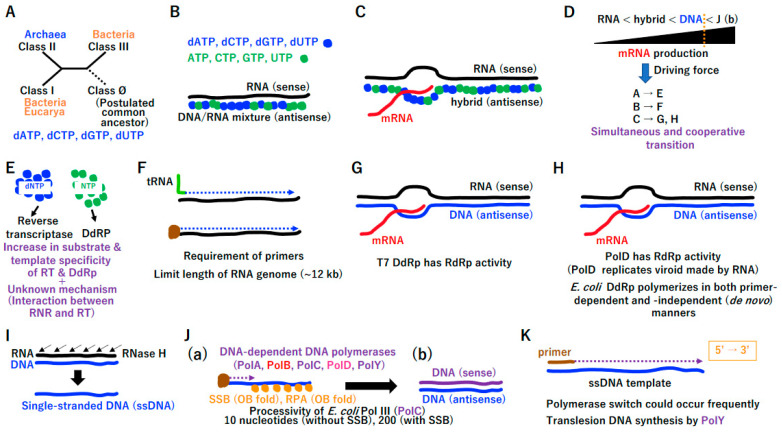
RNA-to-U-DNA transition. (**A**) Ribonucleotide reductase (RNR). Schematic phylogenetic tree of RNR classes I–III. The postulated common ancestor of RNR was reconstructed as class θ. dATP, dCTP, dGTP, and dUTP (not dTTP) (blue characters) could be produced at the chamber of the vent, mediated by RNR. (**B**) Primitive reverse transcription by RdRp (PolB) using template sense RNA and mixed substrates of dNTP (blue) and NTP (green). Black (**F**–**H**), blue (**G**–**K**) and purple (**J**) lines represent RNA, antisense DNA and sense DNA respectively. Red line (**C**,**G**,**H**) represents transcribed mRNA. The two dashed blue (**F**) and purple (**K**) arrows represent DNA synthesis. (**C**) Primitive hybrid strand-dependent polymerization by RdRp (PolB). (**D**) Driving force for the transition from RNA to U-DNA. < indicates the high stability of the antisense strand in a variety of duplexes. (**E**) Selective supply. (**F**) Extant reverse transcriptase (RT) (PolB). (**G**) T7 type DdRp (PolB). (**H**) Bacterial and archaeal DdRps (PolD). Bacterial and archaeal DdRps have a double phi-beta barrel domain (marked as PolD) and polymerize in the 5′ to 3′ direction. (**I**) Generation of single-stranded DNA (ssDNA). Arrows represent degradation of RNA. (**J**) Multiple DNA-dependent DNA polymerases. (**a**) Parallel emergence of multiple DdDps, including PolA, PolB, PolC, PolD, and PolY (Supplementary Figure S4). ssDNA could be covered with single-stranded DNA binding protein (SSB) or RPA. The OB fold, which is derived from the common ribosomal subunit (Supplementary Figure S2A), is common in both SSB and RPA. (**b**) Appearance of the dsDNA single operon. (**K**) Kornberg’s reaction. Low processivity of each DdDP could lead to cooperation rather than competition among multiple DdDp(s) to complete DNA synthesis.

We have previously predicted that a pool of hundreds of single operons associated with both the translation system and extant standard genetic code existed before cells (Supplementary Figure S2) [28]. A small quantity of prebiotically synthesized dNTPs [32] was likely essential for the RNA-to-DNA transition (Supplementary Figure S3), facilitating the cooperative emergence of ribonucleotide reductase (RNR) (Figure 2A) [33], primitive reverse transcriptase (RT) (Figure 2B), and primitive DNA-dependent RNA polymerase (DdRp) (Figure 2C). Extant RNR plays a pivotal role in the synthesis of deoxyribonucleotides from ribonucleotides. This is achieved through the removal of the 2’-hydroxyl group of the ribose ring of nucleoside diphosphates (ADP, GDP, CDP, and UDP), which results in the formation of dADP, dGDP, dCDP, and dUDP. Extant RT is responsible for the conversion of RNA genomes into DNA, a process that is referred to as reverse transcription due to the involvement of RNA-dependent DNA polymerase activity. The emergence of both RNR and RT is essential for the transition from RNA to DNA.

We predict that primitive RT can efficiently utilize NTPs as well as dNTPs. Primitive reverse transcriptase, a derivative of RNA-dependent RNA polymerase, could polymerize the DNA/RNA-mixed antisense molecule (Figure 2B). Primitive reverse transcriptase, which should have the RRM-palm domain, as marked by PolB, has 5′ to 3′ directional polymerization activity. In addition, primitive DdRp could efficiently utilize NTPs as well as dNTPs (Figure 2C). Primitive DNA-dependent RNA polymerase, a derivative of RNA-dependent RNA polymerase, could discriminate DNA/RNA-mixed antisense molecules from RNA (sense molecule), preferentially bind the former, and then polymerize mRNA (sense RNA). Primitive DNA-dependent RNA polymerase, which should have the RRM-palm domain, marked PolB, has 5′ to 3′ directional polymerization activity. Because DNA is a more stable substance than RNA, antisense DNA is an excellent template for multiple rounds of mRNA production (Figure 2D). If the physical stability of the antisense strand in nucleic acid duplexes was beneficial for mRNA productivity, the transition from RNA to U-DNA would inevitably occur.

Among mixtures of dNTP and NTP, both template and substrate specificities could increase gradually in primitive RT (B) and primitive DdRP (C) by the driving force (D). Furthermore, an unknown mechanism could selectively supply corresponding substrates for primitive RT and primitive DdRP (Figure 2E). Finally, antisense nucleotides could transform from RNA to U-DNA. This powerful driving force (Figure 2D) produced extant RT (Figure 2F) together with selective dNTP (Figure 2E), leading to T7 type DNA-dependent RNA polymerase (Figure 2G). All extant RTs have the RRM-palm domain (marked PolB) and possess 5′ to 3′ activity. Although most RTs require tRNA as a primer, protein-primed RTs exist [34,35]. RT is mainly encoded by retroviruses with a genome size of less than 12 kbp (Figure 2F). T7 DdRp has the RRM-palm domain (marked as PolB) and moves in the 5′ to 3′ direction. In addition to antisense DNA, T7 DdRp can synthesize RNA using an RNA template [36] (Figure 2G). Of note, RNA-dependent RNA polymerase (RdRp) (Supplementary Figure S2) has an RRM-palm domain (Supplementary Figure S4). Both RT (Figure 2F) and T7 phage DdRp (Figure 2G) also have the RRM-palm domain (Supplementary Figure S4). In addition to the RRM-palm fold, another DdRP (marked as PolD) derived from the Bacteria or Archaea domains with a double psi-beta barrel domain (Supplementary Figure S4) emerged (Figure 2H) [37]. Since PolD-type DdRp can replicate viroid genomes [38], DdRp uses an RNA template as well as antisense DNA. Utilization of an RNA template by T7 DdRp and bacterial/archaeal DdRP could be reminiscent of primitive DdRp (Figure 2C). *E. coli* DdRp polymerizes in both primer-dependent and -independent manners [39]. The former activity might be related to the gain of PolD-type DNA-dependent DNA polymerase (DdDp).

RNA digestion of RNA/DNA duplexes by RNase H, an essential component of the retrovirus life cycle [40], could lead to single-stranded DNA (Figure 2I). Extant retrovirus encodes RNase H as well as RT. RNase H specifically degrades the RNA of DNA/RNA duplexes [40]. According to the driving force (Figure 2D), ssDNA could be converted into double-stranded DNA (dsDNA) by DNA-dependent DNA polymerases (Figure 2J). To avoid the terminal replication problem, protein-primed-type DNA synthesis [34], as in extant RT (Figure 2F), could have emerged by utilizing a single-stranded DNA binding protein (SSB or RPA type) [41] and DNA-dependent DNA polymerases (DdDps) (Figure 2J(a)). The DdDp PolB (Figure 2J(a)) could be directly derived from T7 type DdRp (Figure 2G), primitive RdRp (Figure 2C), and RdRp (Supplementary Figure S2). To simplify enzyme lineages in evolution, we marked these polymerases as PolB (Figure 2, Supplementary Figure S4). By contrast, DdDp (PolD) (Figure 2J(a)) could be derived from cellular DdRp (Figure 2H), marked PolD. Remarkably, DdDp with another fold (Supplementary Figure S4) could have emerged simultaneously, leading to PolA, PolC, and PolY (Figure 2J(a)). Then, dsDNA might emerge in the LUCA (Figure 2J(b)).

Purified *Escherichia coli*-replicative Pol III (PolC) has a processivity of only 10 or 200 nucleotides in the absence or presence of SSB, respectively [42] (Figure 2J(a)). To complete the DNA replication of a single S10 operon (5.2 kbp) (Supplementary Figure S2B), DdDp should be loaded on the ssDNA template more than 26 (5200/200) times. Thus, a polymerase switch could occur frequently. Since PolY has translesion DNA synthesis activity, the damaged ssDNA template could be converted to dsDNA. Since multiple DdDps, including lesion-bypass-type PolY, could function cooperatively in the completion of DNA replication (Figure 2K), they share similar characteristics depending on the ssDNA template and primer for 5′ to 3′ directional DNA synthesis [42]. These polymerase characteristics were first demonstrated by Kornberg using *E. coli* DNA polymerase I (PolA) [42].

#### 3.1.2. Discrimination of Sense and Antisense DNA

**Figure 3:**
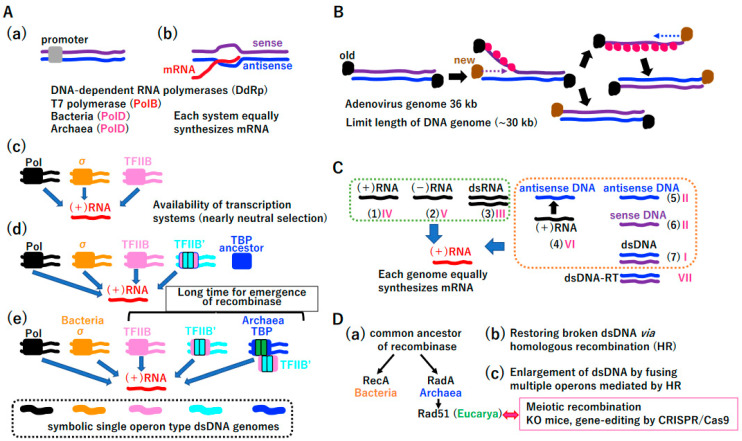
The LUCA—garden of Baltimore. (**A**) Different promoter recognition systems in the era of single operons. (**a**,**b**) Necessity of promoters. Extant T7 and bacterial/Archaeal DdDp can specifically transcribe antisense DNA. (**c**) T7 DdRp directly binds promoters. Bacterial DdRp recognizes the promoter via the σ subunit. Ancient Eucarya (derived from Archaea) DdRp could recognize promoters via ancient TFIIB in early evolution. (**d**) Internal duplication of TFIIB prior to that of TBP. (**e**) The promoter was mainly recognized by TBP. Importantly, any systems described in (**a**–**e**) produce almost the same amount of (+) RNA. Different colored letters and thick lines represent genomes with different transcription initiation mechanisms. (**B**) Strand displacement and unidirectional replication in the single operon era. Black and brown dots represent old and new terminal proteins, required for protein priming. Pink dots represent SSB. (**C**) Garden of Baltimore in the single operon era. Roman numerals I–VII correspond to the classification of viruses by Baltimore [48]. (**D**) Evolution of recombinase (triggering the end of the LUCA). (**a**) Common recombinase emerged in the LUCA. (**b**) HR could restore broken dsDNA using homologous sequences of other DNA (Supplementary Figure S5). (**c**) Fusion of multiple operons in the LUCA. Eucarya Rad51 is the basis for meiotic recombination and biotechnologies.

Since sense and antisense ssDNAs in duplex DNA share the same physical properties, specific RNA synthesis on antisense DNA, as a template, required promoter sequences. Indeed, extant T7 RNA polymerase (PolB) and bacterial/archaeal RNA polymerase (PolD) cannot discriminate sense or antisense strands of duplex DNA without promoters (Figure 3A(a,b)). T7 RNA polymerase directly recognizes the promoter. *E. coli* RNA polymerase recognizes the promoter via σ (Figure 3A(c)). Although Eucarya (derived from Archaea) DdRp recognizes promoters via TBP, a basal transcription factor, it is predicted that TFIIB, another basal transcription factor, initially recognized the promoter. TFIIB in both Achaea and Eucarya interacts with the surface of RNA polymerase, which is structurally equivalent to the σ-interacting surface of *E. coli* RNA polymerase [43,44,45,46]. 

TFIIB may have been the primary promoter recognition protein in the ancient world (Figure 3A(c)) [43,44,45,46,47]. Both extant TFIIB and TBP underwent internal duplication. A phylogenetic analysis of direct repeats revealed that duplication in TFIIB occurred earlier than that in TBP. Thus, TFIIB’ with an internal duplication could recognize the promoter, like the original TFIIB [47]. Although the internal duplication of TFIIB yielded TFIIB′, there was no internal duplication in TBP at that time (Figure 3A(d)) [47]. The role of TBP in transcription initiation cannot be predicted at that time. Extant TBP, the primary site of promoter recognition, has an internal duplication (Figure 3A(e)) [47]. Importantly, various transcription systems (at least five different transcription initiation systems) produce mRNA equally (Figure 3A(c–e)), indicating that weak selection allowed divergence of transcription initiation.

#### 3.1.3. Strand Displacement and Unidirectional Replication

Duplex DNA (up to 30 kbp) could be replicated as follows (Figure 3B). As in extant adenovirus DNA replication, protein-primed 5′ to 3′ directional replication of one template strand occurs, and the other strand is displaced and stabilized by SSB [42,49]. After the replication of one strand is completed, the replication of the other strand occurs (Figure 3B). DNA polymerases and SSB (or RPA) are required for duplex DNA replication, suggesting that DdDp and SSB (RPA) as described in Figure 2J were available. In addition to three types of RNA (Figure 3C (1)(2)(3), Supplementary Figure S2) in the progenote, the LUCA had four types of DNA (Figure 2F,J and Figure 3B,C (4)(5)(6)(7)). Various nucleic acids could directly or indirectly produce almost the same amount of mRNA.

Nucleic acids (1)–(7) in Figure 3C correspond to types I–VI among seven types in a classification of viruses defined by Baltimore [48]. Of note, double-stranded DNA mediated by RT, corresponding to Baltimore class VII, is described in Figure 2D. Considering the diversification of RNR (Figure 2A), multiple DdRps (Figure 2G,H), multiple DdDps and SSBs (RPA) (Figure 2J), multiple transcription initiation systems (Figure 3A), two-types of DNA ligases (Supplementary Figure S5A), and the variety of nucleic acids in the single operon era (Figure 3C), substantial diversification from the LUCA could occur. Thus, the LUCA, as the golden era, can be considered the garden of Baltimore.

The existence of the Ediacaran biome prior to the Cambrian explosion is indicated by fossils found on Earth. It is presumed that the Ediacaran biome fed on mats composed of microorganisms and is therefore called the garden of Ediacaran due to the lack of predators. We propose that a world consisting of a single population of operons (with the genome configuration defined by Baltimore) before the onset of genome gigantism be compared to the garden of Ediacaran and called the garden of Baltimore. The garden of Baltimore is considered identical to the LUCA.

### 3.2. Stage 2 (The End of the LUCA)

Crystallization starts from a seed crystal, which is a small piece of a single crystal. If each single operon-type DNA genome in the LUCA (stage 1) corresponds to a seed crystal, enlargement of crystals will require multiple fusions of single-operon-type DNA genomes. Homologous recombination is a key invention to end the LUCA era. Crossing over-mediated homologous recombination helps two operons to fuse. This means that homologous recombination can quickly create larger and more complex genomic structures, leading to the beginning of genetic linkage (Section 3.2.1), vertical inheritance (Section 3.2.1), and horizontal gene transfer (Section 3.2.2). Thus, the extant genetic system could be established in stage 2.

#### 3.2.1. Beginning of Genetic Linkage and Vertical Inheritance

The balance among single operons encoding various products (Figure 3C) could be rapidly destabilized by an increase in dsDNA due to the gain of the homologous recombination (HR) system, which relies on common recombinases, RecA and RadA (Figure 3D(a)). RecA of Bacteria and RadA of Archaea (the latter derived from Eucarya Rad51) are key recombinases for homologous recombination (HR) [50,51,52]. Since the maximum genome length of extant RNA viruses is 30 kbp (Supplementary Figure S2C), the superior physical nature of dsDNA enabled the generation of genomes exceeding 30 kbp. The evolution of HR to repair errors (restoring broken dsDNA using homologous sequences of other DNA) during DNA replication (Figure 3D(b)) could have been a long process in the robust LUCA (Supplementary Figure S5A,B). In addition, two independent DNA ligases [53] could have emerged to repair template ssDNA with single-stranded breaks (Supplementary Figure S5A(a)). Thus, the basic tool kit for the completion of DNA replication on damaged templates could have been established in the LUCA (Supplementary Figure S5C).

In addition to its repair function, extant HR accelerates a variety of genomic rearrangements, including duplications (Supplementary Figure S6A) [54]. Furthermore, HR greatly accelerates protein evolution (Figure S6B,C,E). Eukaryotic-HR-mediated meiotic recombination prompted species diversification (Figure 3D) [55]. Moreover, biotechnologies, such as knockouts and gene-editing by CRISPR/Cas9, rely on cellular HR (Figure 3D) [56,57]. Thus, HR has been a key event in Darwinian evolution since it arose in the LUCA.

**Figure 4:**
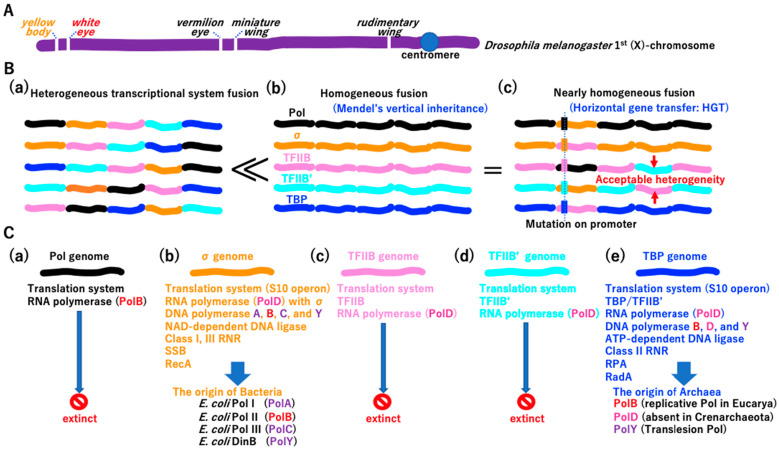
Origin of genetics. (**A**) Well-known example of genetic linkage. Schematic diagram of the *Drosophila melanogaster* 1st (X)-chromosome. Genes affecting a variety of phenotypes are arranged linearly on a single chromosome, as demonstrated by Morgan et al. [58]. (**B**) Early genetics in the fused operon era. Each operon is marked by a different color, representing the transcription initiation system (Figure 3A(e)). << Integrity of (**b**) type fused operons was superior to that of (**a**) type. = if the promoter of the heterogenous operon in the (**c**) type genome could be adapted to surrounding operons by mutation, the integrity of the type (**c**) genome is equivalent to that of the type (**b**) genome. Red arrows indicate acceptable heterogeneity, because TFIIB’ might have similar biochemical activity to that of TFIIB (Figure 3A). (**C**) Persistence of two of five genomes. Although extant cells have either the σ genome (**b**) or TBP genome (**e**), each operated by a σ- or TBP-mediated transcription initiation system (Figure 3A(e)), other genomes (**a**,**c**,**d**) could have also emerged at the LUCA. Stop marks (**a**,**c**,**d**) represent genomes that could not become cells.

Although the *yellow body* phenotype is not biochemically related to the *white eye* phenotype in *Drosophila melanogaster*, the alleles are inherited together due to their linkage on the same chromosome (the same dsDNA molecule) (Figure 4A) [58]. Morgan’s genetic linkage could be the first step in the fusion of two single operons by HR. Transcription initiation could be divided into at least five systems (Figure 3A(e)). HR-mediated fusion of five single operons could lead to heterologous transcriptional system fusion (Figure 4B(a)), homologous transcriptional system fusion (Figure 4B(b)), and nearly homologous transcriptional system fusion (Figure 4B(c)). Homologous transcriptional system fusion could be beneficial over heterologous transcriptional system fusion (Figure 4B).

Since each operon represents a different biochemistry (different phenotype), different phenotypes are in linkage due to the linear arrangement of operons, as in the fly chromosome (Figure 4A). Thus, Morgan’s genetic linkage [58] and Mendel’s vertical inheritance [59] existed since the first operon fusion at the LUCA.

Genes associated with different phenotypes could be genetically linked (Figure 4C). For instance, the σ genome (Bacterial lineage) harbored genes encoding DNA polymerases (Figure 2J), including PolA, PolB, PolC, and PolY, and NAD-dependent DNA ligase (Supplementary Figure S5A(a) and Figure 4C(b)). By contrast, the TBP genome (Archaeal lineage) encoded the DNA polymerases (Figure 2J) PolB, PolD, and PolY and ATP-dependent DNA ligase (Supplementary Figure S5A(a) and Figure 4C(e)). Thus, the difference in transcription initiation easily created the so-called DNA replication divide between Bacteria and Archaea [60]. A network of transcription factors evolved rapidly to regulate biological processes in both σ and TBP genome lineages (Supplementary Figure S7). Extinct genomes not found in free-living cells (Figure 4C(a,c,d)) could also acquire DNA polymerases and DNA ligases in different combinations. We speculated that the initial replication mechanism evolved more than twice in the LUCA. Consequently, *E. coli* (bacteria) has the σ factor and replicative PolIII (PolC). Human cells (Eucarya), descendants of Archaea, have TBP and DNA polymerase α, δ, and ε (all are PolB). Of note, HR-mediated operon fusion ended the single-operon golden era of the LUCA, an active time in the garden of Baltimore.

#### 3.2.2. Beginning of Horizontal Gene Transfer (HGT)

The integration of DNA fragments into the genome by HGT under different transcription initiation systems and subsequent mutations in promoter regions was an important process. As shown in Figure 4, the adaptation of the heterogeneous operon in the surrounding genome (Figure 4B(c)) is the same process as HGT. Thus, HGT began at the first operon fusion (Figure 4B). Over time, beneficial mutations could be transferred among genomes by HGT during evolution.

### 3.3. Stage 3 (Enlargement of the DNA Genome Associated with the “DNA Replication Divide”)

Unlike crystallization of materials, enlargement of DNA genomes corresponding to enlargement of crystals should occur under the physical law of Eigen’s error catastrophe theory [31]. Enlargement of DNA requires sophisticated DNA replication machinery (Section 3.3.1), the DNA repair system (Section 3.3.2 and Section 3.3.4), the uracil-to-thymine transition (Section 3.3.3), and compaction of enlarged DNA genomes (Section 3.3.5). Inventions (Section 3.3.1, Section 3.3.2, Section 3.3.3, Section 3.3.4 and Section 3.3.5) could enable the enlargement of DNA.

#### 3.3.1. Evolution of Two Different DNA Replication Machineries

**Figure 5:**
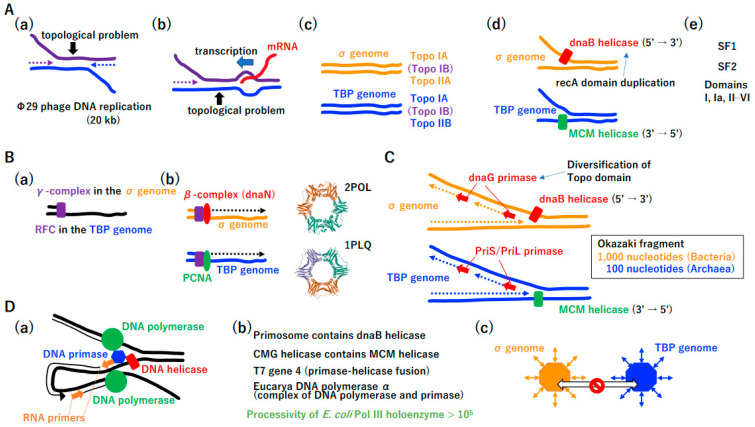
DNA replication divide. (**A**) Overcoming the topological problem. (**a**) Mullis’ reaction. Strand displacement-type DNA replication of ϕ29 phage (20 kbp) occurs simultaneously from both ends [42], as in PCR as invented by Mullis. The speed of the DNA replication of ϕ29 phage is up to twice that of adenovirus DNA replication (Figure 3B). Unwinding from both ends creates tortional stress on the dsDNA region. (**b**) Collision between transcription and replication. During DNA enlargement, transcription and DNA replication should occur simultaneously. The collision of transcription and replication leads to tortional stress on dsDNA. (**c**) Solution to Delbrück’s claim. Type IIA and IIB topoisomerases were invented in the σ genome and TBP genome, respectively. Topoisomerase IA is common to both. Eucarya DNA topoisomerase IB is found in some bacteria and some archaea, suggesting that the development of topoisomerase IB and subsequent HGT across the three kingdoms (Bacteria, Archaea, and Eucarya) occurred. (**d**) Birth of replicative DNA helicase. Topoisomerase offered the opportunity for the high-speed unwinding of dsDNA by DNA helicase. DnaB helicase and MCM helicase were invented in the σ genome and TBP genome, respectively. DnaB helicase arose from the duplication of the recA domain. (**e**) Other helicases. In addition to replicative helicases, a helicase superfamily including SF1 and SF2 arose, and these loci were transferred between the σ and TBP genomes. (**B**) High-speed-leading-strand synthesis. (**a**) Clamp loader. The clamp loaders γ-complex (σ genome) and RFC (TBP genome) are common. (**b**) Clamp. The clamps dnaN (σ genome) and PCNA (TBP genome) are structurally common. 2POL (dnaN) and 1PLQ (PCNA) are PDB IDs. (**C**) Emergence of the lagging strand. Invention of (**A**,**B**) provides a basis for the development of lagging-strand synthesis. DNA primases, dnaG and PriS/PriL, evolved independently in the σ and TBP genomes, respectively. DnaG shares a common domain with topoisomerase, Toprim. Although replication mechanisms are similar in the Bacteria and Archaea domains, the Okazaki fragment in Archaea (including its descendant Eucarya) is shorter than that of Bacteria. (**D**) Coordination of both strands’ DNA synthesis. (**a**) Schematic view of coordinated DNA replication. (**b**) Mutual interactions among replicative proteins. Leading- and lagging-strand synthesis coupled with duplex DNA unwinding are achieved by highly mutual interactions among replicative proteins. DnaB helicase and MCM helicase became components of large protein complexes in the σ and TBP genomes, respectively. In addition, DNA primase and DNA helicase are fused in T7 gene 4. Eucarya DNA polymerase α (PolB) is a complex of DNA polymerase and DNA primase. Although the processivity of *E. coli* PolIII (PolC) is only 10 nucleotides, the processivity of the holoenzyme including PolIII is more than 10^5^ nucleotides. (**c**) Completion of the DNA replication divide. The independent recruitment of replicative proteins (polymerase, helicase, and primase) and highly mutual interactions among replicative proteins led to the complete DNA replication divide between the σ genome (Bacteria) and TBP genome (Archaea) due to the incompatibility of exchanges between equivalent replicative proteins in different lineages.

The replication of large DNA molecules took more time than that required for the replication of small DNA. Accordingly, Mullis-type DNA replication (i.e., the simultaneous replication of both strands) emerged (Figure 5A(a)). Furthermore, replication and transcription occurred simultaneously and collision between these processes should occur occasionally (Figure 5A(b)) [61,62,63,64]. Topological tortional stress has to be increased, as shown in Figure 5A(a,b). After the publication of the DNA duplex model [65], Delbrück claimed that it is impossible to separate dsDNA into two ssDNAs due to a topological constraint [66]; however, the σ genome and TBP genome easily resolved this topological issue by the independent evolution of topoisomerases, TopoIIA and TopoIIB, respectively (Figure 5A(c)) [67,68]. TopoIA is common to both lineages (Figure 5A(c)), which may be explained by HGT. The *TopoIB* gene is distributed in various extant Bacteria and Archaea (Figure 5A(c)), suggesting that TopoIB arose in either Bacteria or Archaea, was transferred to other lineages by HGT, and was inherited by Eucarya.

Resolving topological issues enabled independent origins of dnaB helicase and MCM helicase in each genome (Figure 5A(d)). The dnaB helicase moves in the 5′ to 3′ direction [69]. MCM is translocated in the opposite direction [70]. Of note, dnaB could be derived from the duplication of the recA domain (Figure 5A(d), Supplementary Figure S6C) [71]. Other helicases, including superfamily I and II helicases, may have arisen [72] and spread to both genomes by HGT (Figure 5A(e)). The rapid progression of the replication fork by topoisomerase and helicase enabled high-speed-leading strand DNA synthesis, triggering the evolution of the clamp loader and clamp (Figure 5B(a,b)) [73,74]. Since both the clamp loader and clamp are sequentially and structurally similar in both genomes [75], after they arose in either genome, they could be transferred to the other genome and thereby exert important functions.

High-speed replication fork opening and leading-strand synthesis provided space for lagging-strand synthesis, triggering a two-fold increase in the total replication speed. Under the same driving force, dnaG primase in the σ genome and PriS/PriL primase in the TBP genome could have arisen independently (Figure 5C) [76]. Remarkably, dnaG could be derived from the previously established Topo domain (Figure 5A(c)) [77]. The Okazaki fragment [78] is 1000 or 100 nucleotides in Bacteria or Archaea, respectively (Figure 5C) [79,80].

The coordination of leading- and lagging-strand synthesis (Figure 5D(a)) [81] could be achieved by highly mutual interactions among replicative proteins (Figure 5D(b)). For instance, dnaB helicase and MCM helicase formed large protein complexes, the primosome and CMG (Cdc45-Mcm-GINS) helicase, in Bacteria and Eucarya, respectively [82,83]. In addition to MCM, Cdc45 and GINS [84] are conserved in Eucarya and Archaea. T7 phage gene 4 encodes a primase and helicase fusion protein [85]. Mouse DNA polymerase α is a polymerase and primase complex [86]. Although *E. coli* PolIII has a processivity of only 10 nucleotides (Figure 2J), that of PolIII holoenzyme is more than 10^5^ nucleotides [42]. Thus, the equivalent replication components of the σ genome and TBP genome were no longer exchangeable, leading to a complete DNA replication divide between Bacteria and Archaea (Figure 5D(c)) [60]. Further evolution of DNA replication machineries in the σ genome and TBP genome, based on Jacob’s replicon theory [87], is summarized in Supplementary Figure S8.

#### 3.3.2. Necessity of DNA Repair for the Increase in DNA Genome Size

**Figure 6:**
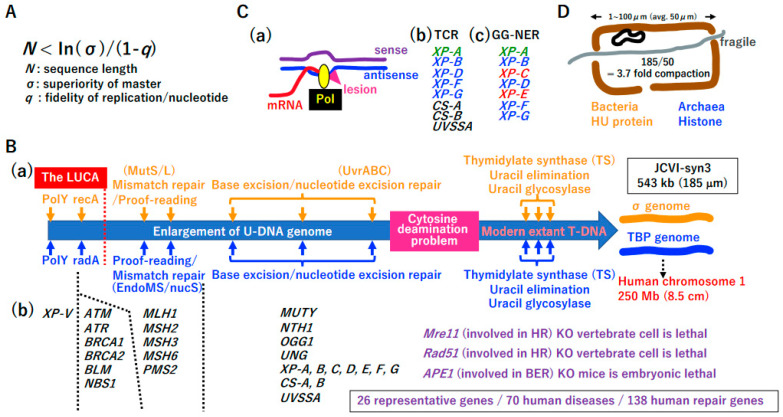
Coevolution of DNA enlargement and the DNA repair system. (**A**) Eigen’s error catastrophe. (**B**) (**a**) Schematic diagram of a proposed mechanism for the evolution of DNA repair. As described in Figure 2J and Figure 3D, translesion synthesis (PolY) and homologous recombination (recA, radA) could be established in the LUCA. Except for base excision repair, DNA repair mechanisms might have evolved independently in the σ or TBP genomes. Toward T-DNA, the cytosine deamination problem should be resolved (see Supplementary Figure S9 for details). Although DNA can increase to up to 8.5 cm (corresponding to human chromosome 1), 185 µm-long T-DNA was expected based on the genome size of the synthetic minimum cell JCVI-syn3. (**b**) Human repair system. A lack of either Mre11 or Rad51, both of which are involved in homologous recombination repair, is lethal in vertebrate cells [98,99]. A lack of Ape1 in mice, a component of BER, leads to embryonic lethality [100]. In addition, 70 of 138 DNA repair-related genes are associated with hereditary diseases in humans [101]. Among these 70 genes, 26 representative genes in the DNA repair pathway of (**B**(**a**)) are listed. (**C**) Hanawalt’s transcription-coupled repair. (**a**) Hanawalt first recognized the existence of transcription-coupled DNA repair (TCR). (**b**) In human cells, XP-B, -D, -F, and -G, all of which are conserved in Archaea, are required for TCR. Since there is no counterpart to the E. coli UvrABC system in Archaea, TCR-NER mainly acts in Archaea. (**c**) Global genome-NER. In human cells, GG-NER could arise from XP-A, -C, and -E, all of which do not exist in Archaea. (**D**) DNA compaction and protection. Elongated and naked dsDNA is physically fragile. Since the predicted average size of chambers in the alkaline vent is 50 µm, the minimum genome size of 185 µm should be compacted and protected by dsDNA binding proteins. HU protein and histone evolved independently in each genome.

According to Eigen’s error catastrophe theory (Figure 6A) [31], it is impossible for the DNA genome to increase in size without the evolution of a DNA repair system. Translesion polymerase (PolY) (Figure 2K and Figure 6B(a)) [88], SSB repair by DNA ligases (Supplementary Figure S5A), and recombinases (Figure 3D and Figure 6B(a)) may have been established in the LUCA (before genome fusion). During genome enlargement, proof-reading of DNA polymerases [42] and a mismatch repair system could have arisen independently in each genome (Figure 6B(a)) [89,90,91]. Enzymes involved in base excision repair (BER) [92,93,94] are largely shared between Bacteria and Archaea, suggesting that genes encoding the enzymes involved in BER in either the σ genome or TBP genome are easily transferred into the other genome by HGT (Figure 4B(c)) [95,96,97].

Although *E. coli* has an efficient nucleotide excision repair (NER) system mediated by UvrABC [102], an NER enzyme is lacking in most species of Archaea. This point will be discussed later (see Figure 6C). Among 138 human DNA repair genes, 70 genes are associated with hereditary diseases [101]. Among these 70 genes, 26 representative genes are listed in Figure 6B(b), such as *XP-V* [103], *NBS* [104], and *XP-A* [105]. Thus, Eigen’s error catastrophe [31], a physical law, has dominantly governed biological processes in all organisms, including humans, since the LUCA era.

#### 3.3.3. Transition from U-DNA to T-DNA

Cytosine often changes into uracil via deamination, leading to mutations during subsequent DNA replication [42]. Extant living cells resolve this problem using the thymine-based DNA genome (T-DNA), rather than the uracil-based DNA genome (U-DNA). This transition simultaneously requires the invention of thymidylate synthetase (TS), uracil elimination by dUMPase, and removal of mis-incorporated uracil by glycosylase (Figure 6B(a)) [42,106]. One scenario for the transition from U-DNA to T-DNA is described in Supplementary Figure S9.

#### 3.3.4. Transcription-Coupled NER

Hanawalt’s group first proposed transcription-coupled repair [107]. RNA polymerase in the elongation step is an excellent sensor of damaged antisense DNA (Figure 6C(a)), and the preferential repair of antisense DNA is highly beneficial for restoring normal transcription. In Eucarya, transcription-coupled NER (TCR-NER) [108] is operated by various proteins, including XP-B, XP-D, XP-F, and XP-G, all of which are conserved in Archaea (Figure 6C(b)). Thus, TCR-NER might mainly function in Archaea to repair DNA damage. It is possible that global genome (GG)-NER in Eucarya (Figure 6C(c)) [109] evolved from TCR-NER via the Eucarya-specific evolution of XP-A, XP-C, and XP-E.

#### 3.3.5. Necessity of DNA Compaction

After the evolution of DNA replication (Figure 5, Supplementary Figure S8) and DNA repair systems (Figure 6B,C), the DNA genome can increase 8.5 cm in length (Figure 6B(a)). Venter’s minimal synthetic cell JCVI-syn3 has a 543 kbp DNA genome [110], with an estimated length of 185 µm. Since the average size of each chamber in a hydrothermal vent (Figure 1B(b) and Figure 6D) is 50 µm, a 185 µm-long DNA genome should be compacted 3.7-fold in the chamber. HU and histone evolution in the σ genome and TBP genome, respectively [111,112], easily solved the compaction problem (Figure 6D).

### 3.4. Stage 4 (Formation of Coacervate, Leading to Proto-Cells with Permeable Membranes)

Enlarged DNA genomes could become new seed crystals (Section 3.4.2), attracting lipids surrounding DNA. Permeable membranes rather than non-permeable membranes could be ideal materials for compartmentalization of each DNA genome (Section 3.4.1).

#### 3.4.1. A Lack of Essential Genes Is Lethal in Extant Cells

**Figure 7:**
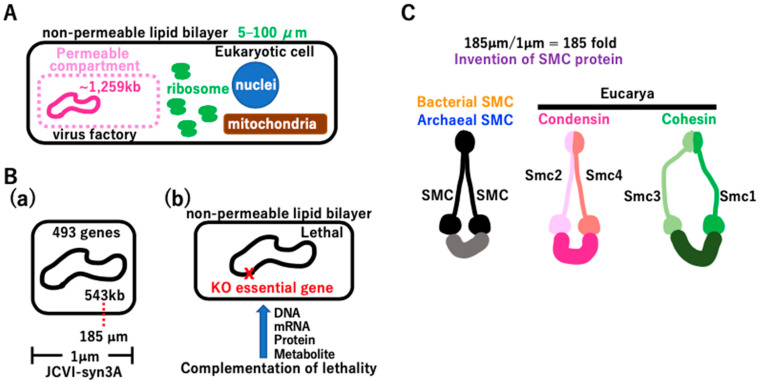
Lethality of a non-permeable membrane surrounding the incomplete genome. (**A**) Giant virus factory. Although the mimivirus has a 1259 kbp genome, larger than of that of JCVI-syn3, it is not free-living. The permeable compartment surrounding the mimivirus genome is called a virus factory. Thus, the permeability of the compartment might be essential for incomplete genomes, such as the mimivirus. (**B**) Incomplete vs. complete genome. (**a**) Synthetic biology (JCVI-syn3A). A compartment surrounded by a non-permeable membrane and cell wall and including a complete genome (543 kbp) leads to free-living JCVI-syn3A cells. (**b**) Cell lethality. If an essential gene is lacking, cell death will occur without complementation of the missing component, similar to experimental results [115,116]. (**C**) Evolution of SMC proteins. Expected size of the first bacteria and archaea might be equivalent to that of JCVI0syn3, 1 μm. Since JCVI-syn3 has 185 µm-long DNA, compaction of more than 185-fold is necessary. SMC proteins could arise in either the σ or TBP genome, followed by transfer to the other genome by HGT. The descendants of prokaryotic SMC in Eucarya are condensin and cohesin.

**Figure 8:**
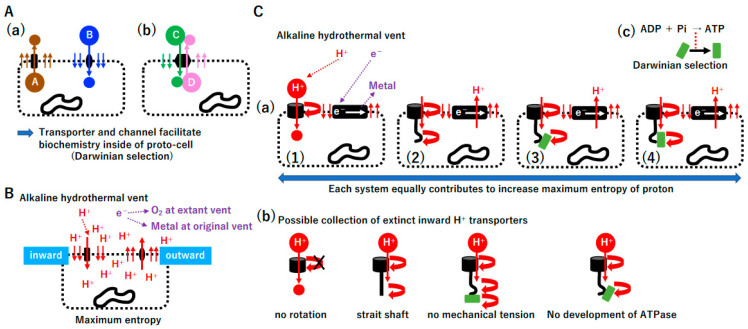
Co-evolution of membrane proteins with permeable membranes. (**A**) Transporter and channel. Large and small closed circles represent concentrations of corresponding substances. Small thin arrows and large thick arrows represent passive and protein-mediated transport. Dashed rectangles represent a permeable membrane encircling an incomplete genome. (**a**) Inward and outward flow of substances. Difference in concentrations between the inside and outside of the proto-cell could create a natural flow of substances via the permeable membrane, resulting in equal concentrations of substances inside and outside of the proto-cell, under the second law of thermodynamics. An outward transporter/channel of substance A and inward transporter/channel of substance B should evolve by Darwinian selection. (**b**) Double transporter. Similar logic to that in (**a**) should result in a double transporter of substances C and D. (**B**) Inward and outward transporter for protons. Small thin arrows and large thick arrows represent passive and protein-mediated proton transport. The extant submarine alkaline vent provides continuous supplies of protons and electrons. A high concentration of protons outside of the proto-cell could result in inward proton transport by Darwinian selection. By contrast, an outward flow of protons could arise by increasing maximum entropy, due to the second law of thermodynamics. (**C**) Birth of ATPase. (**a**) Schematic overview of steps in the evolution of proton-driven membrane-bound ATPase (1)–(4). (**b**) Various types of inward proton transporter/channel/rotator could arise simultaneously in each proto-cell. (**c**) Only extant ATPase persisted because it is extremely useful in producing ATP in the proto-cell and was subjected to powerful Darwinian selection. A possible scenario for the evolution of an electron-transport-coupled outward proton transport system is shown in the Supplementary Materials.

Giant viruses with a 1259 kb genome are not free-living yet are highly active within host cells (Figure 7A). This genome is larger than that of free-living JCVI-syn3 (Figure 7B(a)) [113,114]. The virus factory in Eucarya freely imports and/or exports necessary biomolecules. Thus, an open system is required for the survival of taxa with incomplete genomes, like giant viruses. If extant cells lose a single essential gene, they cannot survive (Figure 7B(b)). For example, the lack of arginine biosynthesis in *Neurospora crassa* leads to cell death [115]. The lethality of *cdc2* mutations in *Schizosaccharomyces pombe* was rescued by the *Homo sapiens* homologue of the *cdc2* gene [116]. As previously reported [117,118], we hypothesized that intermediate proto-cells with an incomplete genome before reaching the free-living state had a permeable membrane to freely exchange necessary biomolecules within the chamber. A variety of membrane-bound proteins could have evolved (see Figure 8 and Supplementary Figure S10). Since the cell size of JCVI-syn3A is 1 µm, the 185 µm DNA genome of JCVI-syn3A should be compacted more than 185-fold (Figure 7B(a),C). Such compaction could be achieved by the SMC protein [119], which could have arisen in either the σ genome or TBP genome, followed by HGT in the other genome (Figure 7C). In Eucarya, condensation compacts metaphase chromosomes [120], and cohesion attaches two sister chromatids [121]. Consideration of Figure 7B,C prompts the question of what type of Darwinian driving force creates proto-cells with permeable membranes and highly compacted DNA genomes.

#### 3.4.2. Coacervate Formation Triggering the Lipid Divide

**Figure 9:**
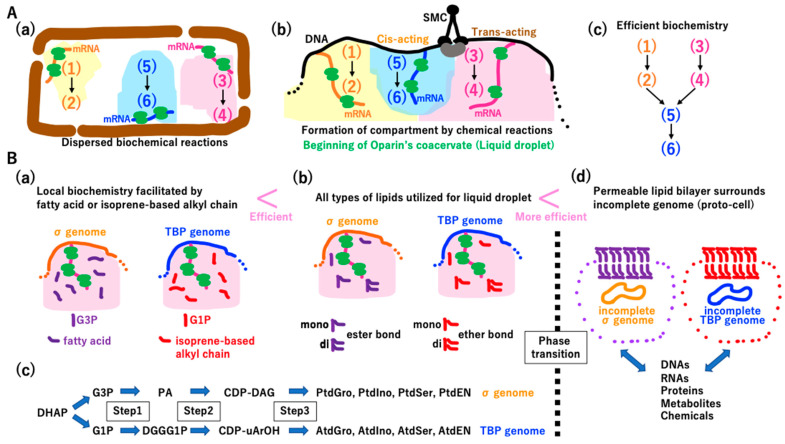
Incomplete large DNA genome surrounded by a permeable membrane. (**A**) Large DNA genome triggering Oparin’s coacervate formation. (**a**) Each operon produces mRNA marked by orange, pink, or blue at a separate location in the chamber of AHV and undergoes its own biochemical reactions (1 to 2, 3 to 4, and 5 to 6) via gene products (enzymes) translated by the ribosome (two tandem green dots represent ribosomal large and small subunits) from individual mRNA. (**b**) Coacervate. Black curved line represents enlarged DNA. It is possible for the orange and blue mRNA-producing DNA regions to be located next to each other (cis-acting) or for the pink mRNA-producing DNA region to be in close proximity to the blue region via SMC (trans-acting). Metabolically related enzymes accumulate in a narrow area, leading to efficient biochemistry due to high concentrations of related metabolites (colored background, corresponding to those in (**a**)). The DNA-mediated hot spot in **A**(**b**) might trigger coacervate formation, originally proposed by Oparin. (**c**) Coacervation facilitates the biochemical network, as extant cells. (**B**) Independent evolution of the synthesis lipid bilayer in a step-by-step manner. (**a**) Fatty acid or fatty alcohol. G3P (glycerol-3 phosphate) and G1P (glycerol-1 phosphate) are derived from the metabolic intermediate DHAP (dihydroxyacetonephosphate) in glycolysis (see (**c**)). Fatty acid or fatty alcohol improve the efficiency of biochemical reactions in the coacervate of **A**(**c**). (**b**) Mono- and di-glyceride of fatty acids and their equivalents in fatty alcohol. All types of lipids could improve the biochemistry in the coacervate. < superior efficiency. (**c**) Lipid divide. DHAP is common in both genomes. σ-genome; G3P, PA(phosphatidic acid), CDP-DAG (CDP-diacylglycerol), PtdGro (phosphatidyl glycerol), PtdInoP (phosphatidyl inositol), PtdSer (phosphatidyl serine), PtdEN (phosphatidyl ethanolamine), and TBP genome; G1P (glycerol-1-phosphate), DGGG-1-P (digeranylgeranyl G1P), CDP-uArOH (CDP-unsaturated archaeol), AtdGro (archaetidyl glycerol), AtdIno (archaetidyl inositol), AtdSer (archaetidyl serine), and AtdEN (archaetidyl ethanolamine) [129]. Genes encoding enzymes in step 1 might have arisen independently in the σ and TBP genomes, followed by vertical inheritance (Figure 4). By contrast, genes encoding enzymes in steps 2 and 3 are common in both genomes [129], suggesting that HGT occurred under the same Darwinian driving force (**A**,**B**) at the chamber of the vent. Thus, the independent invention of enzymes in step 1 in each genome could easily create the so-called “lipid divide” between the Bacteria and Archaea domains. (**d**) Phase transition. < indicates superior efficiency. The physical nature of the di-glyceride of fatty acid or its counterpart in fatty alcohol surrounding the incomplete DNA genome might lead to the formation of proto-cells with a permeable membrane. The incompleteness of proto-cells should require an exchange system for all types of biomolecules.

The reconstructed metabolism of the LUCA is quite similar to that of extant cells [26,27]. A symbolic dispersed biochemical reaction of the LUCA is illustrated in Figure 9A(a). DNA rearrangement and DNA compaction via SMC enabled operons encoding components of three metabolic pathways in Figure 9A(a) to become closer in limited space (Figure 9A(b)), as observed in Eukarya [122]. High concentrations of ribosomes, enzymes, and metabolites could trigger the formation of coacervate [123], also called liquid droplets (Figure 9A(c)) [124,125,126,127,128], leading to efficient biochemistry (Figure 9A(c)).

The lipid divide between Bacteria (σ genome) and Archaea (TBP genome) [129] can be explained by equal contributions of fatty acids and fatty alcohols to the biochemistry of coacervate (Figure 9B(a)). Moreover, if di-acyl-glycerol is a better biochemical facilitator than mono-acyl-glycerol, which is a better biochemical facilitator than either glycerol or fatty acids (Figure 9B(b)), the σ genome could obtain all the biochemical steps one-by-one (Figure 9B(c) upper column). A similar scenario (Figure 9B(b)) could occur independently in the TBP genome (Figure 9B(c) lower column).

Simultaneously and independently, a phase transition could happen in both σ and TBP incomplete genomes, leading to the development of a permeable membrane (Figure 9B(d)). Importantly, if the hypotheses described in Figure 9 are correct, the enlargement, rearrangement, and proper compaction of the DNA genome, enabling better biochemistry, will inevitably produce proto-cells in the chamber. Then, both the replication divide and lipid divide between Bacteria and Archaea could be fully completed at this stage (Figure 9B(d)).

### 3.5. Stage 5 (Evolution of Membrane Proteins on Permeable Membranes and the Beginning of “Omnis Cellula a Cellula”)

Membrane proteins, which help to support biochemistry inside compartments, could emerge under the second law of thermodynamics and Darwinian selection (Section 3.5.1 and Section 3.5.2). Furthermore, the second law of thermodynamics and Darwinian selection could facilitate the emergence of a proto-cell proliferation system in an “omnis cellula a cellula” manner (Section 3.5.3). Of note, each proto-cell (established in stage 5) could become the next seed crystal (see stage 6).

#### 3.5.1. The Second Law of Thermodynamics in Transporter and Channel Creation

The coevolution of the membrane and membrane-bound proteins was proposed [130]. We hypothesize that such coevolution occurs under a combination of the second law of thermodynamics and Darwinian selection. Under the second law of thermodynamics, a high concentration of substance A inside a proto-cell moves outward through a permeable membrane; then, to improve this flow, transporter A from the inner to outer space evolved by Darwinian selection (Figure 8A(a)). Similarly, transporter B and dual transporters C/D emerged. Since the proto-cell produces substance A and consumes substance B, such transporters could facilitate metabolism in proto-cells substantially.

#### 3.5.2. ATPase and the Electron Transport System

Protons and electrons are supplied to the chambers of extant alkaline vents (Supplementary Figure S1B) [18,19,131,132,133,134,135,136,137]. External protons move into proto-cells through the permeable membrane, accumulate in proto-cells, and move outside, reaching maximum entropy by the second law of thermodynamics (Figure 8B). Under a combination of the second law of thermodynamics and Darwinian selection, both inward and outward transporters (channel) of protons inevitably emerged (Figure 8B). A variety of inward and outward proton transporters likely arose (Figure 8C, Supplementary Figure S10). Importantly, all proton transporters contributed equally to the improved current under a combination of the second law of thermodynamics and Darwinian selection. However, among these, proton-current-driven ATPase (Figure 8C(c)) and outward proton pumps coupled with electron transport (Supplementary Figure S10B(e)) were positively selected due to the benefits with respect to energy and biochemistry for proto-cells. After the ATPase or electron transport system arose in either the σ or TBP genome, the corresponding gene could be transferred into the other genome and retained due to its important function. In addition to the transport of small molecules, incomplete genomes also require macromolecule transport mechanisms. As hypothesized previously [138,139], a proposed ancient membrane-based macromolecule exchange system is summarized in Supplementary Figure S11.

#### 3.5.3. Omnis Cellula a Cellula

**Figure 10:**
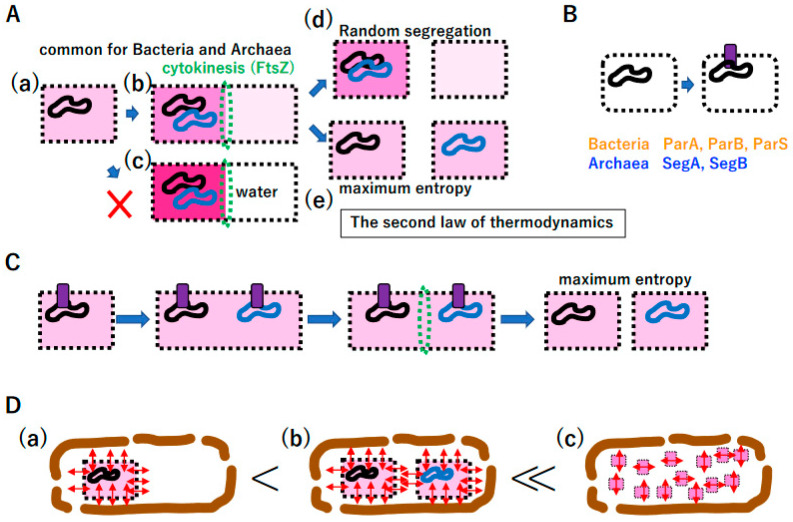
Omnis cellula a cellula. (**A**) Evolution of the lipid bilayer. (**a**) Proto-cell. (**b**) The proto-cell contains a duplicated genome and large lipid bilayer. Although cytokinesis of the enlarged proto-cell might occur naturally, FtsZ (green dashed circle), common to both the σ and TBP genome, could arise in accordance with Darwinian selection. (**c**) Extremely unequal cytokinesis. Complete unequal cytokinesis including all macromolecules and small molecules (difference in the background pink color in left and right compartments) is counter to the second law of thermodynamics. (**d**) Random segregation. If one daughter cell and the other inherit two or no copies of the genome, some unequal components between daughter cells remain. (**e**) Equal genome segregation. Different from the random process in (**d**), equal genome segregation could maximize entropy within two daughter cells. Thus, equal proto-cell division could evolve under the second law of thermodynamics. (**B**) Evolution of genome anchoring to the lipid bilayer. Purple rectangle represents a membrane-bound protein anchoring genomic DNA. (**C**) Origin of Virchow’s “Omnis cellula a cellula”at the proto-cell. After DNA replication, duplicated DNA segregation could arise (Supplementary Figure S8C). An increasing lipid bilayer intrinsically divides (**A**(**b**–**d**)). Thus, concerted effects of duplicated DNA separation, the anchoring of each genome to lipids, and increasing membrane size could yield equal proto-cell division. Importantly, this proto-cell division might maximize entropy in the two daughter cells. (**D**) “Omnis cellula a cellula” in the chamber driven by the second law of thermodynamics. The red arrows at both ends show the entropy increase due to free and active diffusion of various substances. (**a**) As shown in Figure 8 and Supplementary Figure S10, each proto-cell could maximize inner and outer entropy. (**b**) Furthermore, equal proto-cell division could increase entropy in two daughter cells. (**c**) Thus, increasing numbers of proto-cells in a manner consistent with “omnis cellula a cellula” could maximize the total entropy in the chamber of the vent. Then, “omnis cellula a cellula” could become deeply and genetically engrained in the σ and TBP genomes.

In research of prebiotic proto-cells, a vesicle self-reproduction process has been proposed [140]. The continuous supply of lipids to membrane vesicles in vitro inevitably leads to vesicle division due to the physical nature of the lipid bilayer [126]. Based on the physics of the lipid bilayer, FtsZ-mediated cytokinesis [141,142] inevitably arose (Figure 10A(a,b)). Since the concentration of all molecules on one side after cytokinesis is counter to the second law of thermodynamics (Figure 10A(c)), after proto-cell division, equal segregation of chromosomes yields maximum entropy in the production of two daughter proto-cells (Figure 10A(d,e)). Since pathways for the unlinking of replicated DNA were already established (Supplementary Figure S8C) and membrane-vesicle division is an intrinsic property of a lipid bilayer [126], the development of anchoring DNA on the membrane enabled the equal segregation of duplicated DNA genomes into daughter cells (Figure 10B); subsequently, ParA, ParB, and ParS in the σ-genome and SegA and SegB in the TBP genome could emerge (Figure 10B) [143].

Coordination among DNA replication/segregation, membrane growth, anchoring DNA to the membrane, and cytokinesis could establish primitive proto-cell division (Figure 10C). Importantly, the equal division of proto-cells into daughter cells maximizes entropy within the proto-cell (Figure 10C). Moreover, the proliferation of proto-cells in a manner consistent with “omnis cellula a cellula” [144] maximizes total entropy in the chamber as well as entropy in each proto-cell (Figure 10D). Thus, the second law of thermodynamics could promote the establishment of extant living cells, following “omnis cellula a cellula”.

### 3.6. Stage 6 (The End of Proto-Cells with Permeable Membranes and the Beginning of Non-Free-Living Cells with Non-Permeable Membranes)

Although large crystals made from materials can be cracked into two pieces, two large crystals never fuse. Unlike crystals made from materials, proto-cells (intermediate crystal [stage 5] compared with the first seed crystal [stage 2]) could fuse and split due to the physical nature of DNA and lipids and the biological requirement (Darwinian selection) (Section 3.6.1). Then, a proto-cell as an intermediate crystal could repeatedly fuse and split (Section 3.6.1), leading to a much larger crystal (proto-cell containing a nearly complete genome and a non-permeable membrane). Such an enlarged proto-cell could initiate a parallel-crystallization process of proto-bacteria and proto-archaea and another crystallization process of a selfish genome (later becoming viruses and mobile elements) (Section 3.6.2).

#### 3.6.1. Punctuated Equilibrium by the Fusion of Different Systems

Gould proposed the concept of punctuated equilibrium based on fossil data [145]. In the history of life on our planet, the symbiosis of Archaea and Bacteria (Figure 1A(a), Supplementary Figure S12A(a)(2)) and the symbiosis of Eucarya and Cyanobacteria (Figure 1A(b), Supplementary Figure S12A(a)(3)) can be described as examples of punctuated equilibrium at the cellular level. Moreover, another example of punctuated equilibrium, the fusion of photosystem I and II, could yield Cyanobacteria (Supplementary Figure S12A(a)(1)), which produced oxygen and changed the environment of the whole planet.

**Figure 11:**
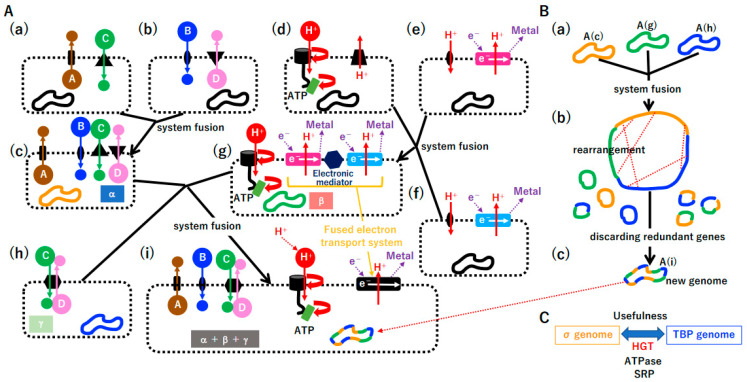
Gould’s punctuated equilibrium at the proto-cell level. (**A**) Punctuated equilibrium. The putative lipid vesicle fusion (including proto-cell fusion) system in the ancient world is described in Supplementary Figure S11. Among proto-cells, proto-cell (**c**) was derived from the fusion of (**a**,**b**), and the proto-cell (**g**) was from (**d**–**f**). In proto-cell (**g**), two independent electron transport systems (marked in pink and sky blue) could fuse due to the invention of an electron mediator (hexagon). The fusion of proto-cells (**c**,**g**,**h**) could yield proto-cell (**i**). Of note, the dual transporter for substances C and D in proto-cell (**h**), instead of the independent transporter for substance C or D in proto-cell (**c**), could be selected by Darwinian selection in proto-cell (**i**). The ideal combination of membrane-bound proteins and combined biochemistry (α, β, and γ) could be achieved. (**B**) Genome rearrangement. (**a**–**c**) During the fusion steps of (**A**(**c**)), (**A**(**g**)), and (**A**(**h**)), each genome fusion, rearrangement, and loss created a new genome for proto-cell (**A**(**i**)). (**C**) HGT. Beneficial genomic elements arising in either the σ or TBP genome could be transferred to the other genome by HGT. Membrane-bound proteins, ATPase, and SRP are common to the Bacteria and Archaea domains, suggesting that HGT occurred in an ancient chamber of the vent.

Considering that free-living cells derived from giant viruses did not arise for 2 billion years (Supplementary Figure S12(b)) [113], punctuated equilibrium by system fusion likely occurred in the chamber of the original alkaline vent (Figure 11) during a relatively narrow time window (Supplementary Figure S12A(a)). Since we hypothesize that membrane–vesicle fusion and the split system were present at the chamber (Supplementary Figure S11), a cascade of proto-cell fusion could rapidly create new and complicated proto-cells in a pattern of punctuated equilibrium (Figure 11A). During proto-cell fusion, DNA rearrangement by HR could create new genomes (Figure 11B). Once useful protein machinery arose in either the σ genome or TBP genome, DNA encoding such machinery could be transferred into the other genome by HGT (Figure 11C). For instance, ATPase and SRP are common in the Bacterial and Archaea domains [26,130,146]. By a cascade of proto-cell fusion (Supplementary Figure S13A) associated with genomic rearrangement (Supplementary Figure S13B(a,b)), proto-cells carrying a nearly complete σ genome or TBP genome may have simultaneously arisen, after which the permeable membrane could be transformed into a non-permeable membrane (Supplementary Figure S13B(c)).

#### 3.6.2. Selfish Cells Trigger the Emergence of Selfish Genes

**Figure 12:**
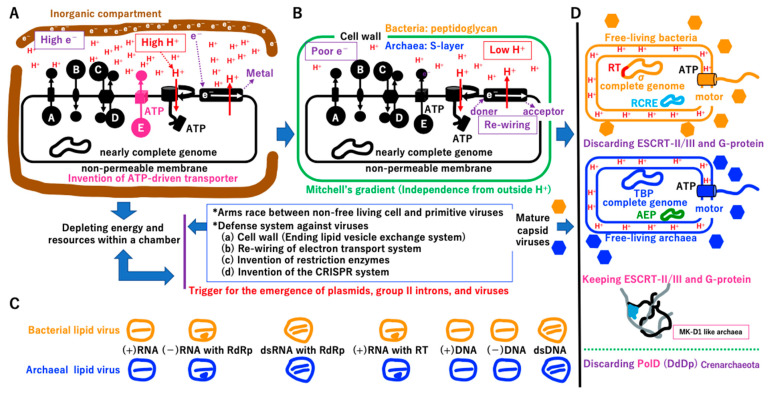
Crystallization of systems in bacteria, archaea, viruses, and mobile elements. (**A**) Living cell with a nearly complete genome and non-permeable membrane. Living cells dependent on the proton supply from the inorganic chamber of the vent could obtain enough ATP to develop an ATP-driven transporter for substance E. Living cells might start to occupy all areas of the original vent, depleting energy and resources. These events could trigger a mass extinction of intermediates from the primitive cell to proto-cell (Figure 2, Figure 3, Figure 4, Figure 5, Figure 6, Figure 7, Figure 8, Figure 9, Figure 10 and Figure 11). During the mass extinction, surviving intermediates might transform to proto-viruses (see Supplementary Figure S14C). One of the most deadly proto-viruses could be made by lipid vesicles. (**B**) Cell wall. The asterisks represent the major changes (**a**–**d**) that occurred in the cells as a result of arms race. An arms race between proto-cells/non-free-living cells and proto-viruses could strongly drive the formation of a cell wall of non-free-living cells (**A**). Although a cell wall could prevent the entrance of enveloped viruses (**C**), an arms race could continue between cells and capsid-based viruses with DNA or RNA [150,151]. The arms race could drive the evolution of restriction enzyme and CRISPR defense systems in cells and a DNA injection system of capsid viruses. Cell wall formation could limit the utility of the external proton and external electron supply. Formation of a cell wall could strongly drive re-wiring of the electron transport system (Supplementary Figure S10B), resembling the extant one. Then, non-free-living cells could survive using their own energy-generating system, enabling the emergence of free-living cells. (**C**) Possible extinct proto-viruses based on lipids. Seven types of nucleic acids encoding replicases enveloped by a lipid bilayer could exist in an ancient world. (**D**) Crystallization. Systems in the Bacteria and Archaea domains with their viruses could undergo crystallization simultaneously at the original vent. Motor protein complexes embedded in the cell wall could arise in both lineages [148], leading to free-swimming bacteria and archaea. Mobile elements, such as plasmids encoding RCRE (pale blue) and AEP (green) (Supplementary Figure S4), could be crystalized mainly in bacteria and archaea, respectively (see Figure 13B). Mobile elements integrated in the bacterial genome could encode RT (red). Since ESCRT-dependent vesicle transport/cell-fusion (Supplementary Figure S11) might not be necessary for both bacteria and archaea, the ESCRT system could be abandoned in both lineages. Some archaea, including MK-D1, could retain the ESCRT system for intracellular vesicle transport. Ancestral archaea ((**D**) upper archaea marked in blue) might have had both PolB and PolD DNA polymerases. In Crenarchaeote, the duplication of PolB and loss of PolD might have occurred during evolution.

Primitive living cells could develop active transporters using ATP created by ATPase (Figure 12A) and start to use all energy and resources in the chamber in a selfish manner, as do extant bacteria and archaea. Thus, all intermediates described in Figure 2, Figure 3, Figure 4, Figure 5, Figure 6, Figure 7, Figure 8, Figure 9, Figure 10 and Figure 11, including proto-cells with incomplete genomes, would go extinct (Supplementary Figure S14A, see Figure 12A). Small genes encoding replicases, such as RdRp, RT, and DdDp, could become selfish DNA as in primitive viruses, and an arms race between selfish cells and selfish replicators is possible (Figure 12A,B). Most deadly viruses would be enveloped because the membrane–vesicle-mediated macromolecule transport system supported metabolism in the chamber (Supplementary Figure S11E). Furthermore, primitive viruses could infect proto-cells as well as primitive living cells, just like mimivirus (giant virus) infection by virophages (Supplementary Figure S14B) [147]. Thus, proto-cells should be affected by a shortage of energy and resources as well as viral infection, leading to rapid extinction in the original vent.

### 3.7. Stage 7 (Crystallization)

The final crystallization processes could simultaneously produce bacteria, archaea, viruses, and mobile elements in the original AHV (Section 3.7.1). It is hypothesized that the arms race gave rise to free-living cells and the viruses that infected them. Non-free-living cells were able to obtain energy by attaching themselves to the chamber of the AHV. However, in order to evade persistent assaults from the primordial virus, they developed a cell wall as a defensive mechanism against its invasion. The cell wall effectively eradicated membrane-bearing viruses yet simultaneously impeded the acquisition of energy from AHVs. In order to surmount this obstacle, a reaction system was devised to generate energy independently of AHV, culminating in the emergence of cells capable of autonomous survival. While the cell wall served as a barrier against viral invasion, viruses evolved specialized penetration mechanisms, resulting in the diverse array of viruses that exist today. Of note, the products of final crystallization (free-living cells with viruses) could spread across the entire planet (Section 3.7.2).

#### 3.7.1. Independent Cell Wall Formation

To prevent the entry of enveloped viruses into primitive cells (Figure 12A), a cell wall may have evolved independently in primitive cells containing the σ or TBP genome (Figure 12B) [148]. The cell wall would be an intrinsically double-edged sword for the bacteria and archaea. The endless supply of protons and electrons from the vent (Figure 12A) would not be accessible. However, re-wiring of electron transport from the system in Figure 12A to that in Figure 12B could support survival of cells with a cell wall. Thus, the coupling of phosphorylation to electron and hydrogen transfer by a chemiosmotic mechanism [149] could be established (Figure 12B), enabling the emergence of free-living cells at the original alkaline vent. Additionally, cell fusion (Figure 11) and membrane-mediated transport (Supplementary Figure S11E) would no longer be possible.

By contrast, primitive cells (Figure 12A) would no longer depend on the inorganic chamber of the original vent (Figure 12B). The cell wall could eliminate enveloped viruses (Figure 12C); however, capsid-based viruses would remain [150]. A flagellin motor could be anchored to the solid cell wall [152,153]), leading to free-swimming bacteria and archaea (Figure 12D).

Genes encoding the postulated ESCRT-mediated membrane–vesicle transport system (Supplementary Figure S11E) would be lost in all bacteria and most archaea (Figure 12D) due to lack of necessity. Similarly, an archaeal lineage, *Crenarchaeote*, has two copies of PolB and could discard PolD [154] due to the redundancy of DNA polymerases (Figure 12D, Supplementary Figure S4). Extant slow-growing archaea (with a doubling time of one month) with an elongated, branched form [155] maintain the ESCRT system for internal vesicle trafficking (Figure 12D, Supplementary Figure S11D). Thus, ESCRT in some archaea might be a molecular fossil remain of an ancient extinct membrane-vesicle transporting system at the chamber of the original vent (Figure 12D, Supplementary Figure S11E). Restriction enzymes [156] and the CRISPR/Cas9 system [57] could have evolved during an arms race between capsid-based viruses and their hosts (Figure 12A,B, Supplementary Figures S14 and S15).

#### 3.7.2. Why Is the Genetic Code Universal?

**Figure 13:**
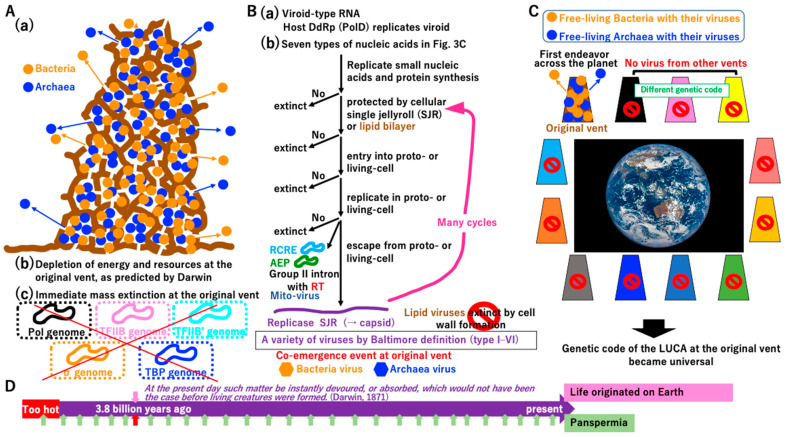
Explanation for the universal standard genetic code. (**A**) Mass extinction at the original vent. (**a**) The original alkaline vent might have been occupied by free-living bacteria (orange closed circle) and archaea (blue closed circle). The orange and blue arrows represent free-living bacteria and archaea, respectively, that have been escaped from the original vent. (**b**) Event (**a**) might exhaust all energy and resources at the vent. (**c**) Events (**a**,**b**) might lead to the mass extinction of most of intermediates described in Figure 2, Figure 3, Figure 4, Figure 5, Figure 6, Figure 7, Figure 8, Figure 9, Figure 10 and Figure 11. This would reduce the activity of the proto-cell, which is completely dependent on such energy and resources at the vent. The color of each genome is the same as in Figure 4C. A large red cross sign means extinction. (**B**) Purification of survivors. (**a**) Extant viroid RNAs can be copied by host DNA-dependent RNA polymerase. Thus, ancient viroid-type RNAs could survive a mass extinction (**A**). (**b**) During the exhaustion of energy and resources at the vent, small nucleic acids might be replicated to avoid extinction. The remaining nucleic acids could be protected by the single jellyroll protein (SJR), which is derived from the proto-cell and later became a capsid protein, or by a lipid bilayer to prevent extinction. Nucleic acids covered with SJR or a membrane might enter living cells. Nucleic acids in cells could replicate. Thus, nucleic acids should encode replicase. Replicated molecules might remain in cells, leading to mobile elements. The extant mobile elements encode RCRE (blue) or RT (red) in the Bacteria domain and AEP (grren) in the Archaea domain (Supplementary Figure S4), as observed in the mitovirus found in some extant mitochondria. Nucleic acids covered with SJR or lipids could escape from cells, leading to proto-viruses. During many infection cycles as described in (**A**,**B**), proto-viruses could rapidly evolve to become deadly viruses. Since cell wall formation (Figure 12B), enveloped viruses might be lost due to an inability to enter cells. Stop marks (**B,C**) indicate extinction. Systems in ancestors of extant viruses could be crystalized at the original vent. The orange and blue closed hexagons represent the bacterial and archaeal lineage viruses, respectively. (**C**) Mass extinction on the planet. Bacteria and archaea with their corresponding viruses might leave the original vent (brown) and spread widely, eventually inhabiting all alkaline vents (a variety of colors) similar to the original one (brown). Since other vents might use different genetic codes, process (**B**) might not produce viruses at these other vents. Thus, the genetic code at the original vent could become universal. (**D**) Against panspermia. Living cells with viruses were brought to the Earth 3.8 billion years ago according to the panspermia hypothesis (red arrow) (lower column), raising the following question: why has other life (many green arrows) not been brought to the planet? Thus, it is likely that life originated on the Earth through the process described in Figure 2, Figure 3, Figure 4, Figure 5, Figure 6, Figure 7, Figure 8, Figure 9, Figure 10, Figure 11, Figure 12 and Figure 13, 3.8 billion years ago (upper column).

Free-living bacteria and archaea at the original vent would lead to the mass extinction of intermediates (Figure 13A(a–c), Supplementary Figure S14). During the depletion of energy and resources at the original vent, viroid-type RNA [38] would survive (Figure 13B(a)). Small nucleic acids of any of the seven types summarized in Figure 3C could be synthesized and protected by the single jellyroll (SHR) protein derived from the proto-cell [150] or the membrane (Supplementary Figure S11E). The evolutionary process described in Figure 13B could rapidly create a variety of viruses. Since cell wall formation in primitive cells (Figure 12B) could eliminate enveloped viruses, only capsid-based viruses would remain at the original vent.

Plasmids encoding RCRE (PolB) or AEP (PolB) and mobile elements encoding RT (PolB) (Supplementary Figure S4) cannot escape from host cells, just like the mitovirus [157]. We speculated that these plasmids and mobile elements lost capsids or were introduced from a primitive enveloped virus. In the latter case, cell wall formation in host cells could permanently trap RCRE, AEP, and RT (Figure 12D and Figure 13B). The remaining nucleic acids not existing at the time of the LUCA (Baltimore definition class VII) (Figure 3C) [48] appeared in the σ genome as mobile elements (i.e., group II introns) (Figure 12D and Figure 13B).

When bacteria and archaea with their viruses migrated from the original vent to the ocean, cells with viruses occupied all alkaline vents on the planet. Although intermediates described in Figure 2, Figure 3, Figure 4, Figure 5, Figure 6, Figure 7, Figure 8, Figure 9, Figure 10 and Figure 11 could be present at some vents, they would eventually go extinct (Figure 13C), as in the original vent (Figure 13A). Gene flow between taxa and populations at different vents was not possible (Figure 13C), because the genetic code likely varied among vents [28], leading to the standard genetic code of the progenote (Supplementary Figure S2) and the LUCA (Figure 3) from the original vent and its global spread (Figure 13C).

According to Darwin’s statement, “At the present day such matter (Events in Figure 1, Figure 2, Figure 3, Figure 4, Figure 5, Figure 6, Figure 7, Figure 8, Figure 9, Figure 10, Figure 11 and Figure 12) be instantly devoured, or absorbed, which would not have been the case before creatures were formed” [158] (Figure 13D), we predict that the crystallization event only occurred once at the original vent 3.8 billion years ago (Figure 13D, upper column). Although an alternative evolutionary theory, panspermia [159], has been proposed repeatedly [160], it does not effectively explain why bacteria and archaea reaching the Earth 3.8 billion years ago grew, while other taxa did not survive (Figure 13D, lower column).

## 4. Conclusions

**Figure 14:**
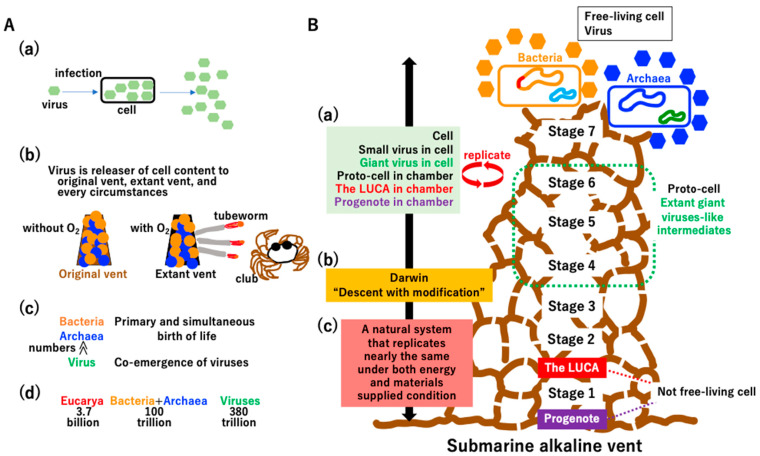
Definition of life. (**A**) Two opposite perspectives. Bacteria and Archaea are marked by orange and blue, respectively. (**a**) Viruses are parasites of living cells. (**b**) Cells are parasites at the original vent and extant vents. Extant vents (brown) are occupied by bacteria (closed orange circle) and archaea (closed blue circle). Tubeworms and other taxa in the extant vent (black) are completely dependent on such prokaryotes. Since extant viruses release cell contents to the extant vent, ancient viruses could release cell contents to the original vent. (**c**) Virosphere. Number of viruses exceeds that of cells. (**d**) Number of viruses in the human body. (**B**) Definition of life. A schematic diagram of the crystallization of processes is presented on the right. It is proposed that the evolution from progenote to LUCA, proto-cell and free-living cell occurred in AHV through the steps from stage 1 to 7 described in this study. (**a**) Replicator. In addition to free-living bacteria and archaea, other intermediates, including small and large viruses, proto-cells, the LUCA, and progenote, were not free-living. Red mutually circular arrows mean replication. (**b**) Darwin’s word. (**c**) Definition of life, including (**a**) all.

Although viruses are believed to be parasites of host cells (Figure 14A(a)), the first free-living cells would likely be selfish parasites of the original vent (Figure 14(b)). Massive numbers of viruses may have been produced from the LUCA [161] at the original vent (Figure 13A(a) and Figure 14A(c)). Moreover, there are far more viruses than taxa belonging to the Bacteria, Archaea, and Eucarya domains in the virosphere (Figure 14A(c)) [162] or cells on the human body (Figure 14A(d)) [163]. Thus, viruses should be included in the definition of life [164,165].

Key innovations for evolution from the progenote to free-living cells with viruses (Figure 14B, right cartoon of the vent) are summarized in Supplementary Figure S15. Kornberg stated “*I have never met a dull enzyme*” [166], and key enzymes and proteins forming the extant biological world have been never dull since they emerged at the original vent (Supplementary Figure S15).

Some evolutionary steps described in Figure 7, Figure 8, Figure 9, Figure 10 and Figure 11 and Supplementary Figures S10–S13 can be tested in vitro and/or in silico. For the testing of the hypotheses of this study, possible in vitro and in silico experiments are presented in Supplementary Figure S16, as follows: synthetic cells with U-DNA can be established in vitro (Supplementary Figure S9); liquid droplets nucleated around compacted DNA can be formed in vitro (Figure 9); vesicles with a permeable membrane and extant membrane bound proteins under the endless supplies of electrons, protons, and resources can be reconstructed in vitro (Figure 8); the principal of “omnis cellula a cellula” in the chamber of the alkaline vent can be evaluated by in silico experiments under the second law of thermodynamics (Figure 10); a postulated vesicle transport system in the ancient vent (Figure 11 and Supplementary Figure S11) can be evaluated by in silico experiments; and artificial fusion among proto-cells containing an incomplete genome could yield free-living cells in vitro (Figure 11).

Finally, although the progenote (Supplementary Figure S2), the LUCA (Figure 3), and intermediates were not free-living (Figure 4, Figure 5, Figure 6, Figure 7, Figure 8, Figure 9, Figure 10, Figure 11 and Figure 14B), all extant life including cells, viruses, and mobile elements are clearly descendants of these non-free-living materials. Cells, viruses, the LUCA, and progenote commonly replicate (Figure 14B(a)). Although Darwin articulately described life as “*descent with modification*” (Figure 14B(b)) [167], this statement could apply to man-made artifacts, such as smartphones, personal computers, televisions, and cars. Thus, we propose that life, including cells, viruses, mobile elements, the LUCA, and progenote, can be defined as “a natural system that replicates nearly the same under both energy- and material-supplied conditions” (Figure 14B(c)).

## Data Availability

All data are included in this article and the Supplementary Materials.

## Supplementary Materials

The following supporting information can be downloaded at https://www.mdpi.com/article/10.3390/biology14010003/s1, Figure S1: Submarine alkaline hydrothermal vent; Figure S2: Progenote; Figure S3: Darwinian selection on the transition from RNA to U-DNA; Figure S4: Variety of replicases; Figure S5: Establishment of homologous recombination; Figure S6: Recombination-mediated genome rearrangement; Figure S7: Transcriptional regulation; Figure S8: Establishment of Jacob’s replicon; Figure S9: Possible scenario for the transition from U-DNA to T-DNA; Figure S10: Electron transport system; Figure S11: Postulated ancient vesicle transport system; Figure S12: Punctuated equilibrium at the cellular level; Figure S13: Emergence of a nearly complete genome with a non-permeable membrane; Figure S14: Cell wall formation and the extinction of enveloped viruses; Figure S15: New proposal for the crystallization process in the origin of bacteria, archaea, viruses, and mobile elements; Figure S16: Possible in vitro and in silico experiments to test the hypotheses presented in this study.
