# Supplementary material for: The Possible Crystallization Process in the Origin of Bacteria, Archaea, Viruses, and Mobile Elements
Source: Biology (Basel). 2024 Dec 24;14(1):3. doi: 10.3390/biology14010003 (PMC11763024; doi:10.3390/biology14010003)
Supplement: Supplementary file 1 [file biology-14-00003-s001.zip › biology-3314486-supplementary.pdf]

Supplementary Materials

The Possible Crystallization Process in The Origin of Bacteria, Archaea, Viruses, and Mobile Elements

Akari Yoshimura and Masayuki Seki

1. **Supplementary figures**
2. **Supplementary figure legends**
3. **Supplementary references**

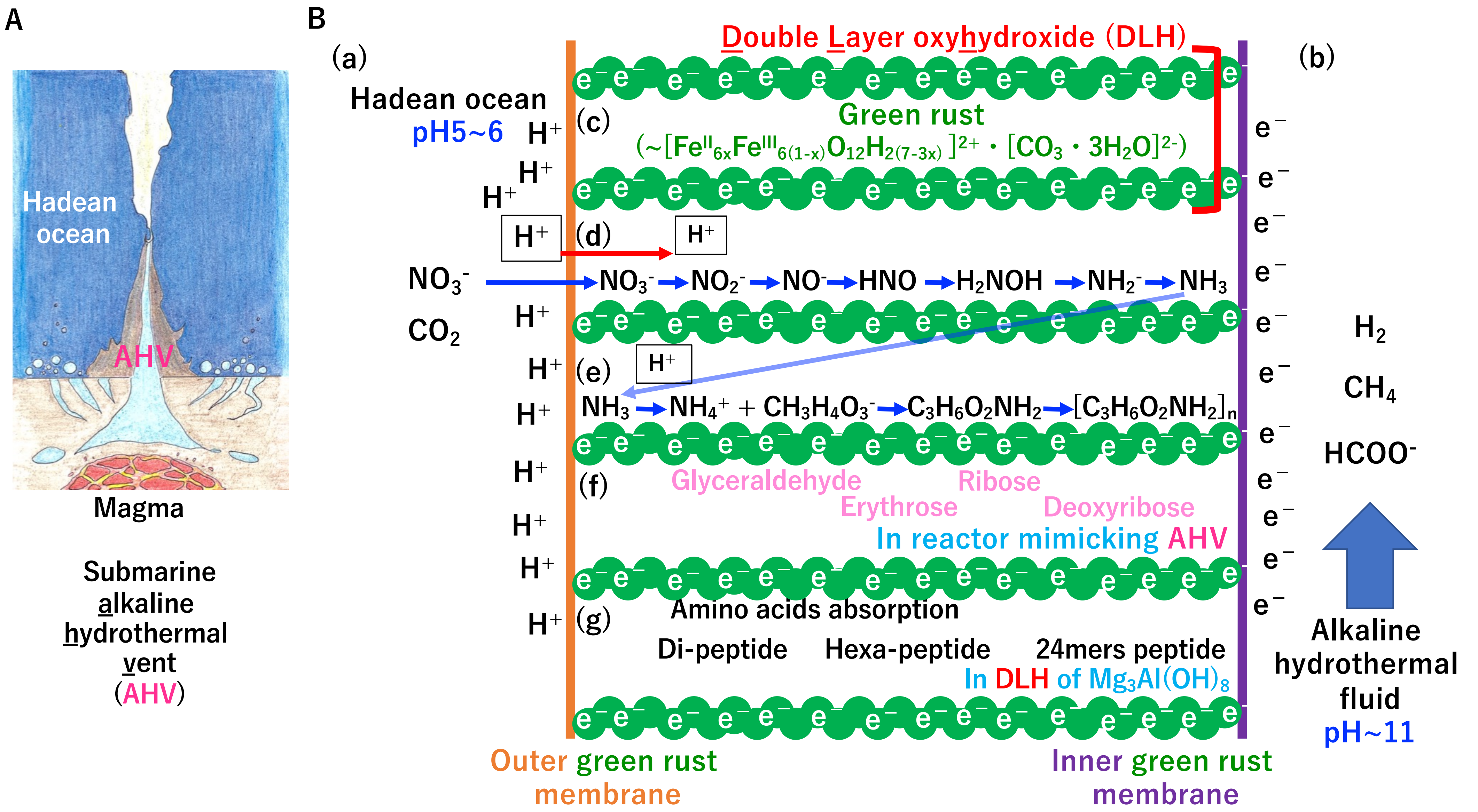

Supplementary Figure S1

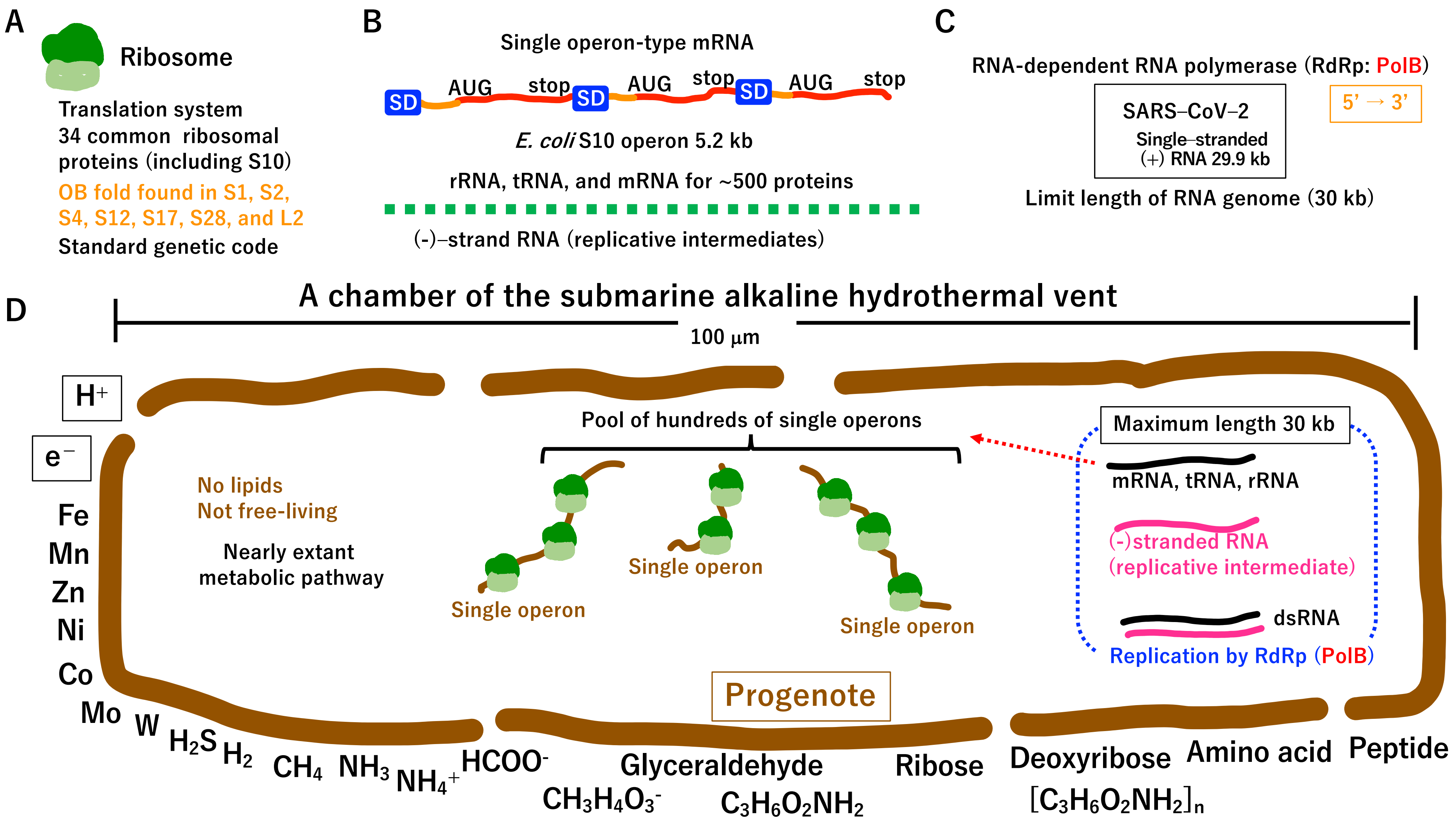

Supplementary Figure S2

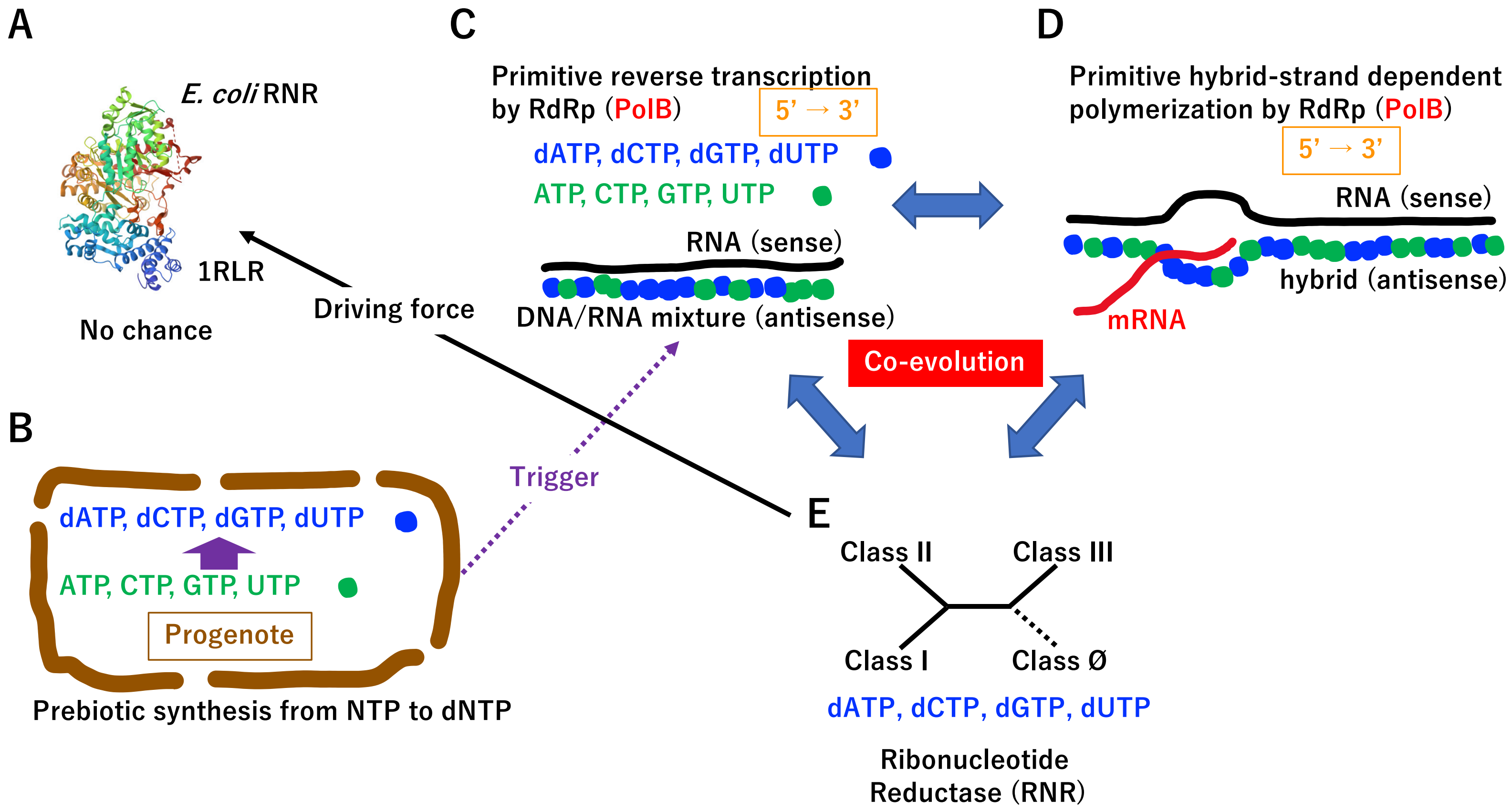

Supplementary Figure S3

	Characteristic domain	Bacteria	Archaea		*H. sapiens*	Mobile element	Virus
		E. coli	*Eury-archaeota*	*Cren-archaeota*			
RNA-dependent RNA polymerase (RdRp) (PolB)	RRM palm	–	–	–	–	–	YES
Reverse transcriptase (RT) (PolB)	RRM palm	–	–	–	–	Group II intron	YES
T7 DNA-dependent RNA polymerase (DdRp) (PolB)	RRM palm	–	–	–	–	–	YES
DNA-dependent RNA polymerase (DdRp) (PolD)	Double psi-beta barrel	DdRp	DdRp	DdRp	Pol I, II, III	nd	YES
Type A DNA-dependent DNA polymerase (DdDp) (PolA)	RRM palm	Pol I	–	–	γ : mitochondria<br>θ : nuclear	–	YES
Type B DdDp (PolB)	RRM palm	Pol II	DdDp	DdDp	α δ ε ζ	YES	YES
Type C DdDp (PolC)	PolC-like	Pol III	–	–	–	nd	nd
Type D DdDp (PolD)	Double psi-beta barrel	–	DdDp	–	–	nd	nd
Type X DdDp	Loop1	–	–	–	β λ μ TdT	–	nd
Type Y DdDp (PolY)	Little finger	DinB UmuC	YES	YES	η ι κ Rev1	nd	nd
Archaeo-eukaryotic primase (AEP)	RRM palm	–	–	–	PrimPol	Plasmid	YES
Rolling-circle replication endonuclease (RCRE)	RRM palm	–	–	–	–	Plasmid	YES

Supplementary Figure S4

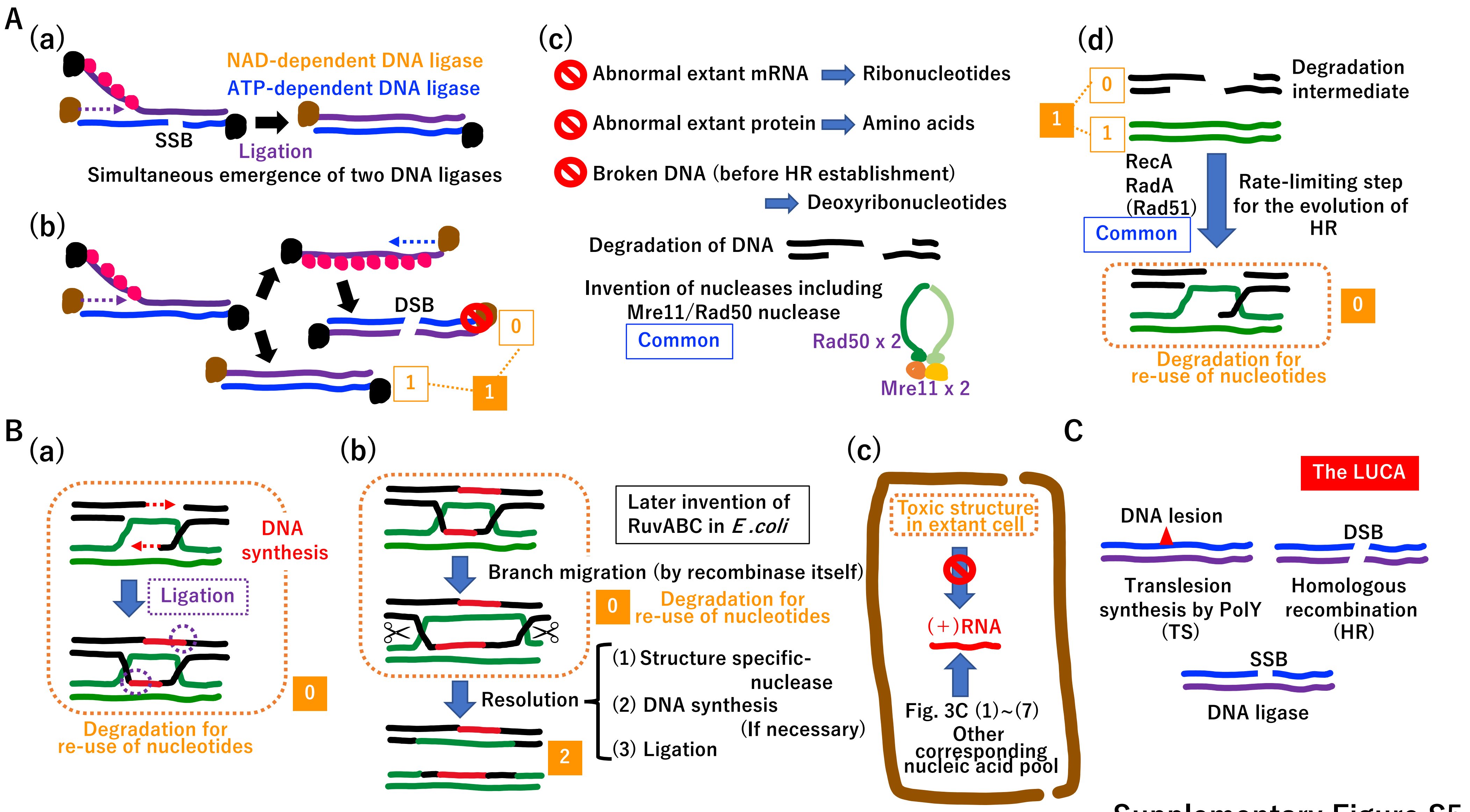

Supplementary Figure S5

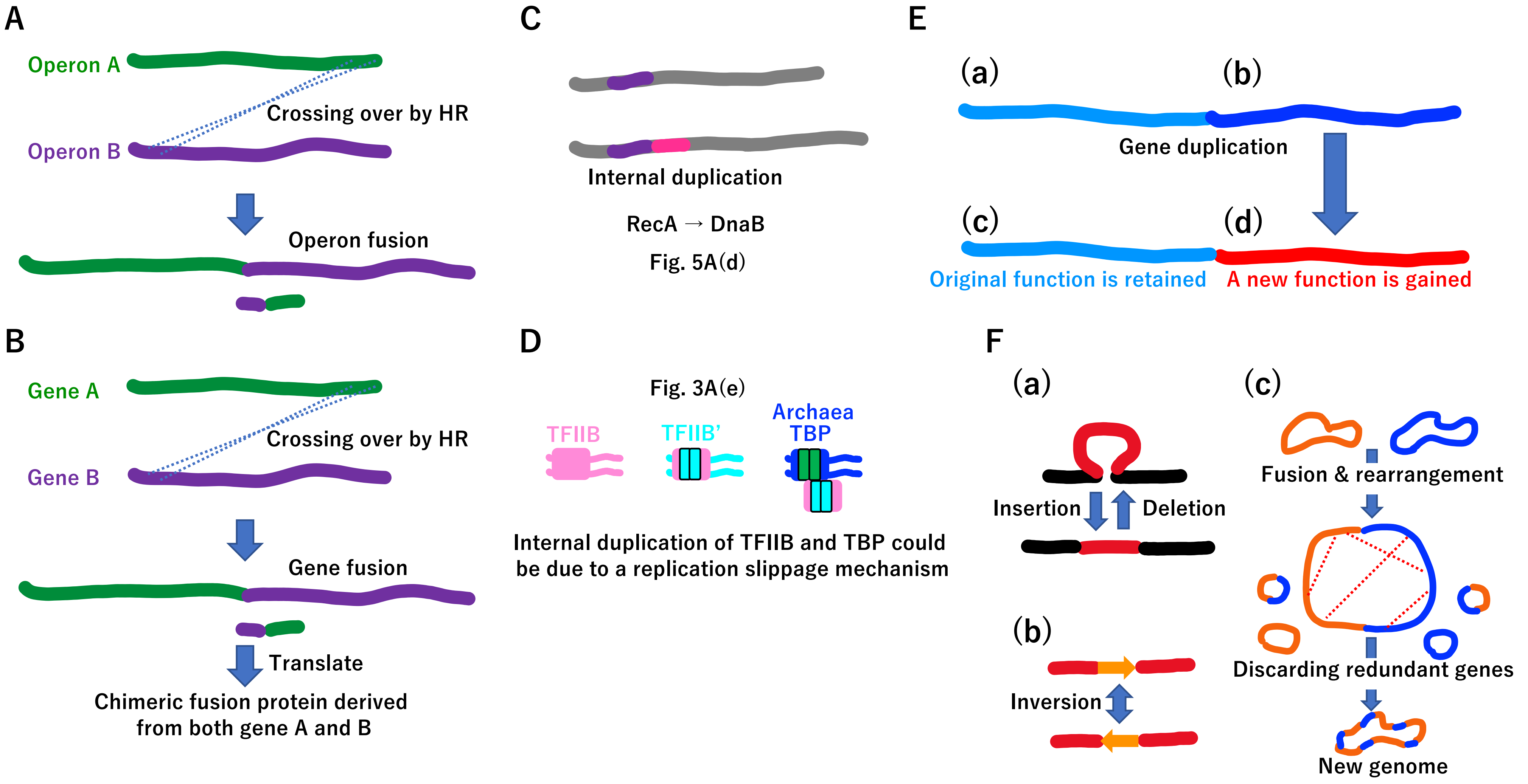

Supplementary Figure S6

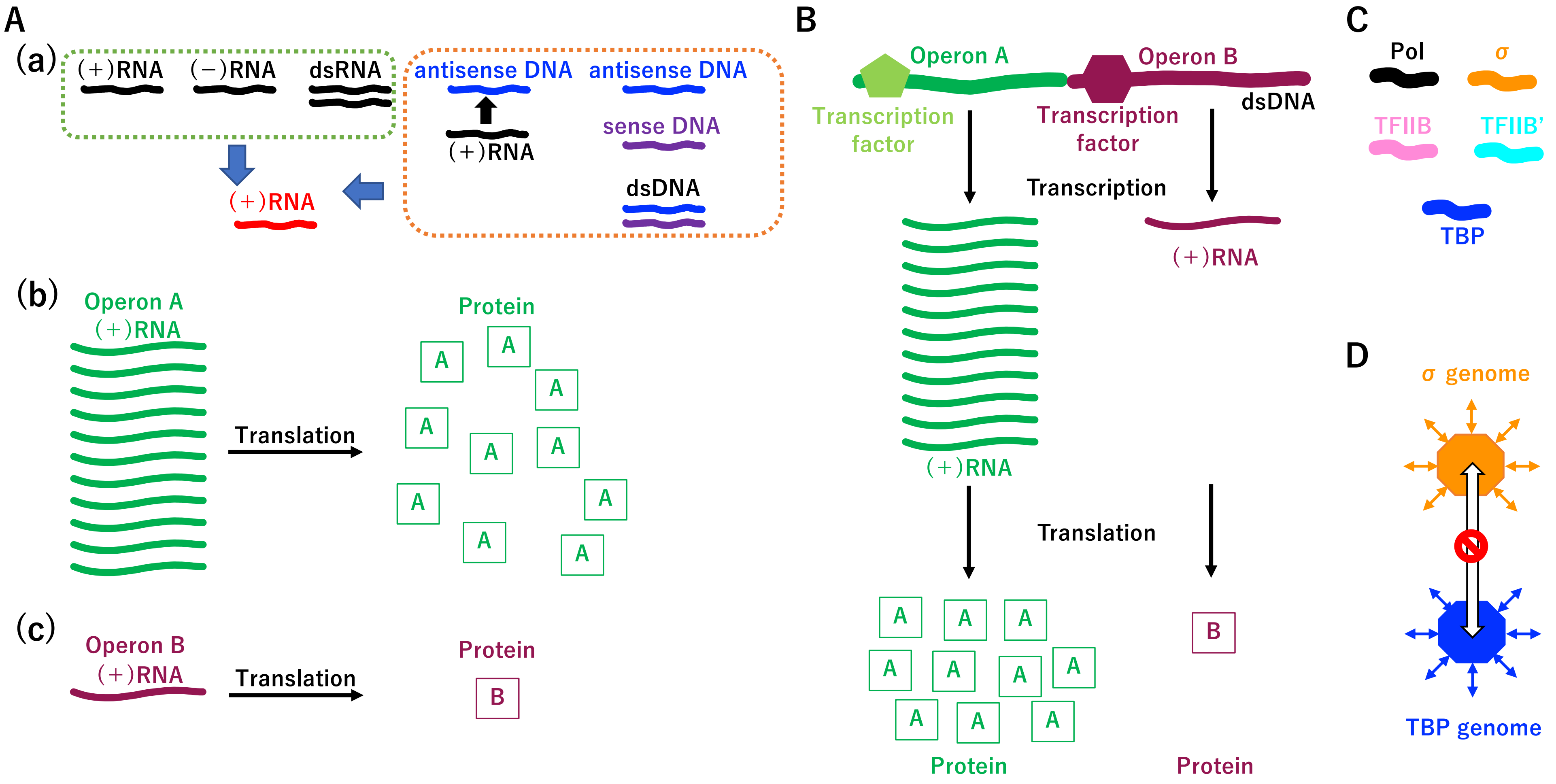

Supplementary Figure S7

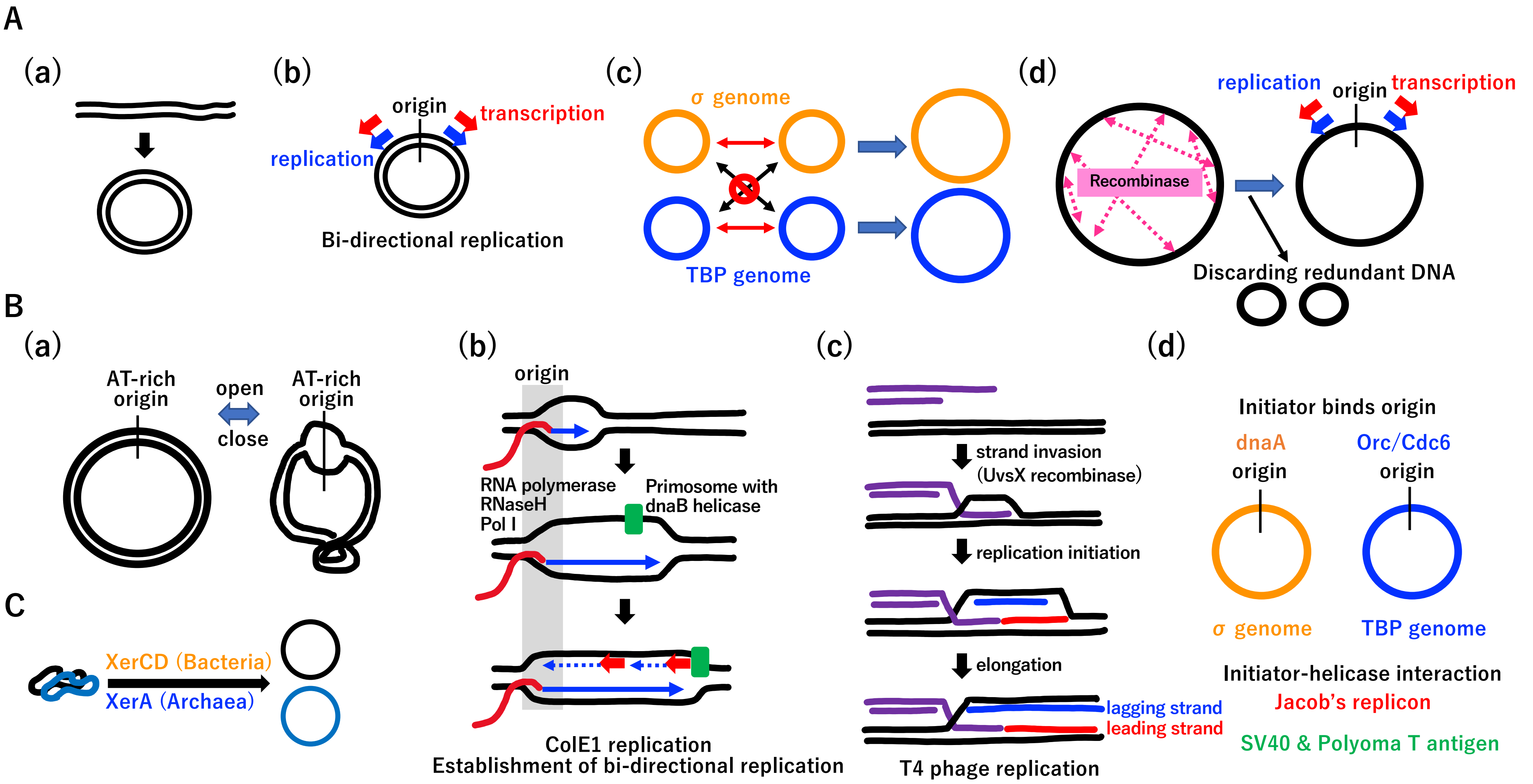

Supplementary Figure S8

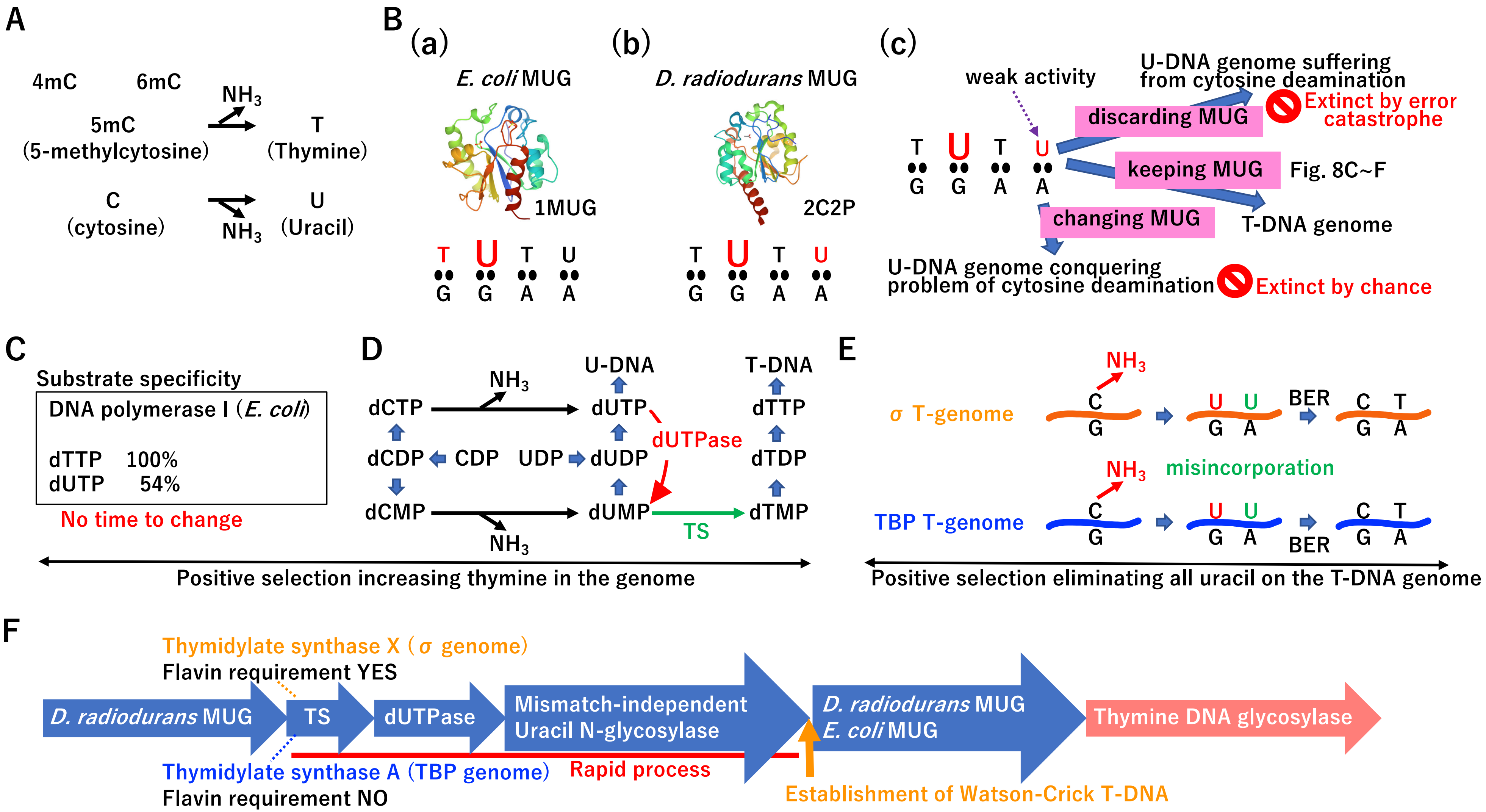

Supplementary Figure S9

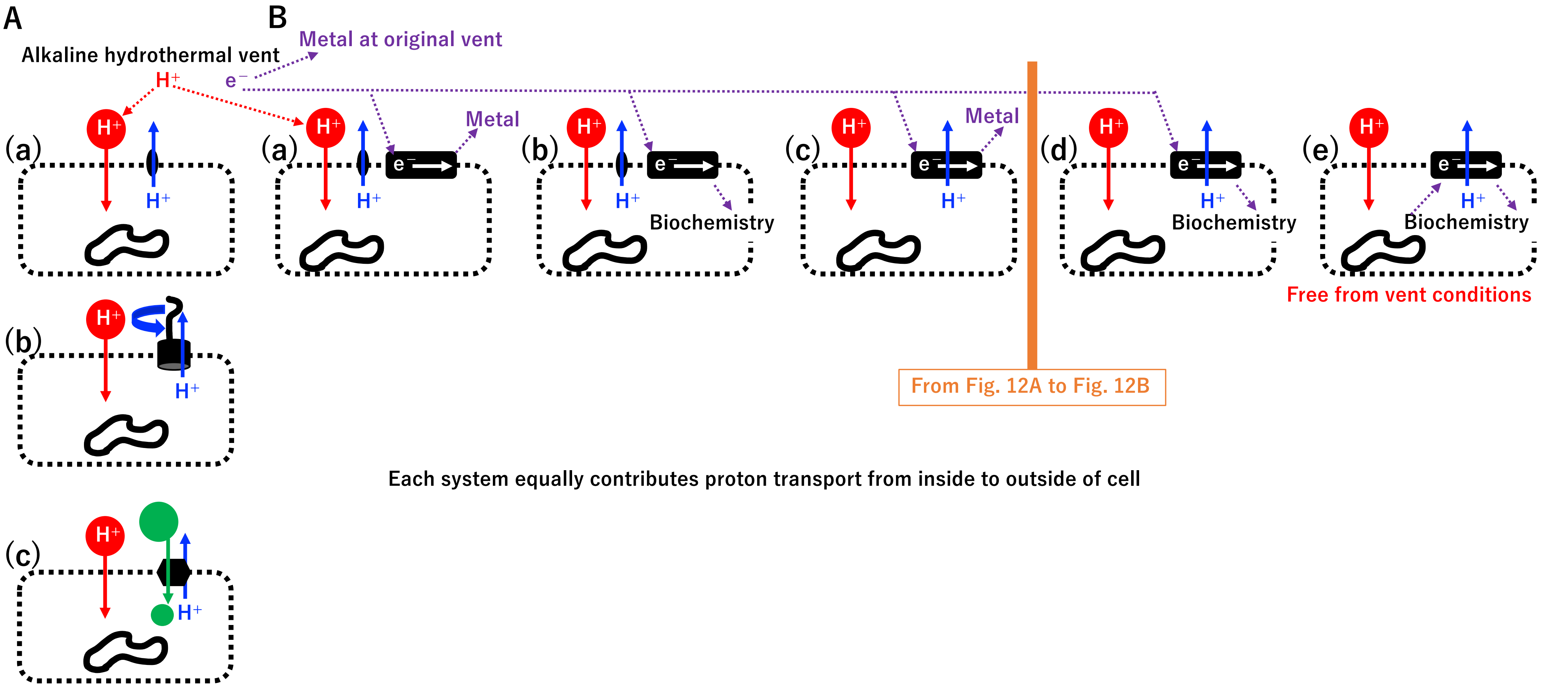

Supplementary Figure S10

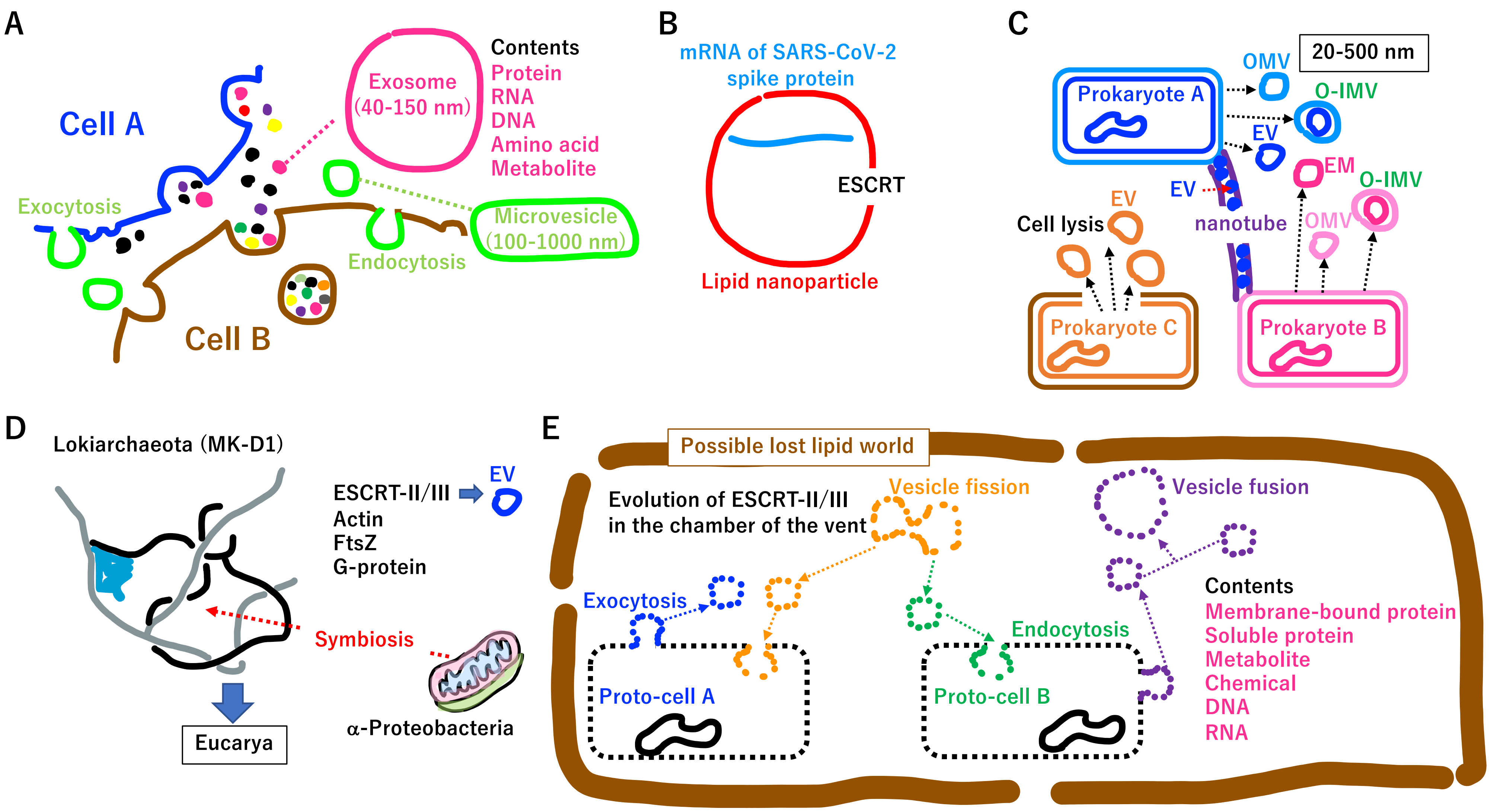

Supplementary Figure S11

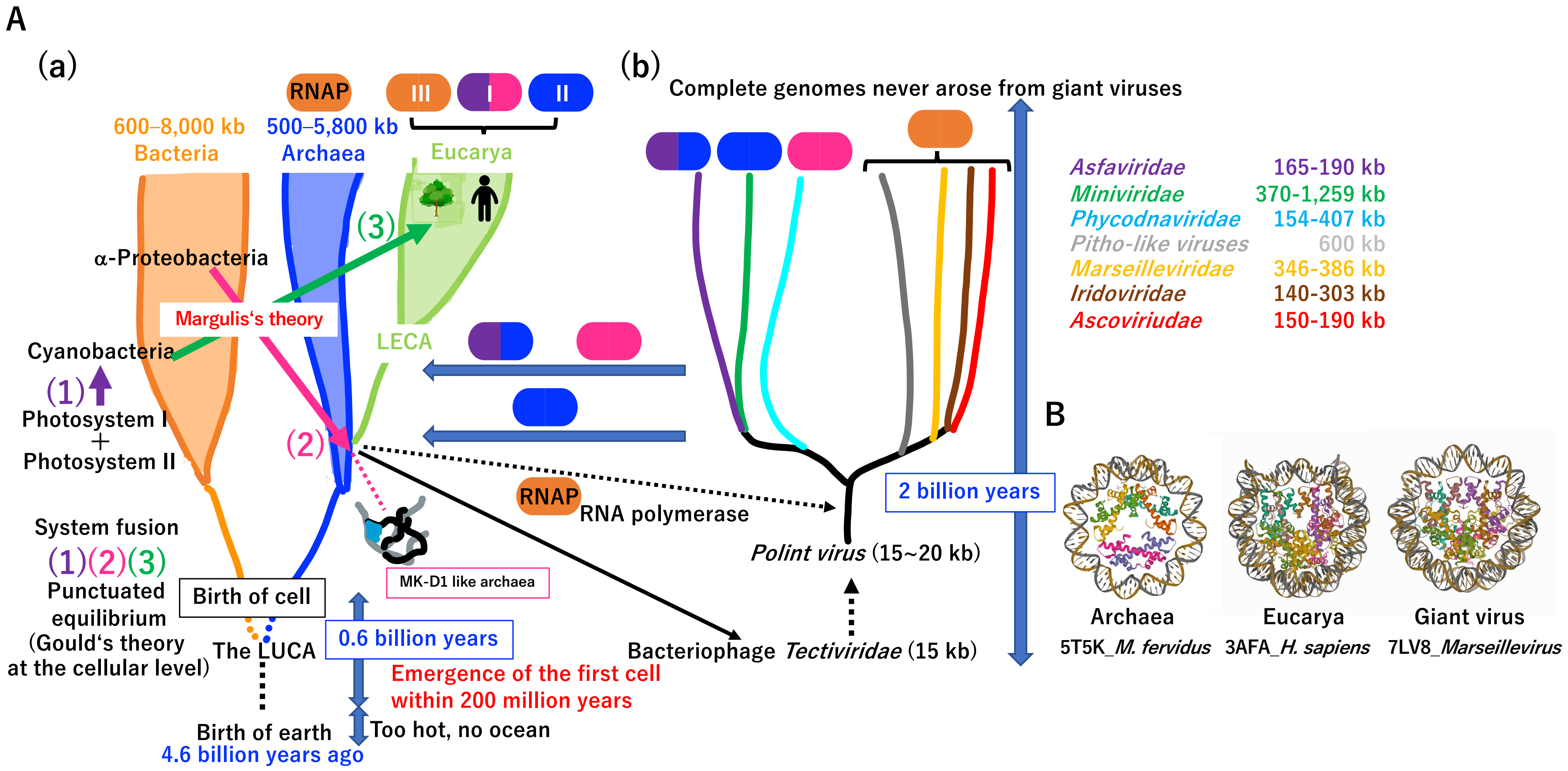

Supplementary Figure S12

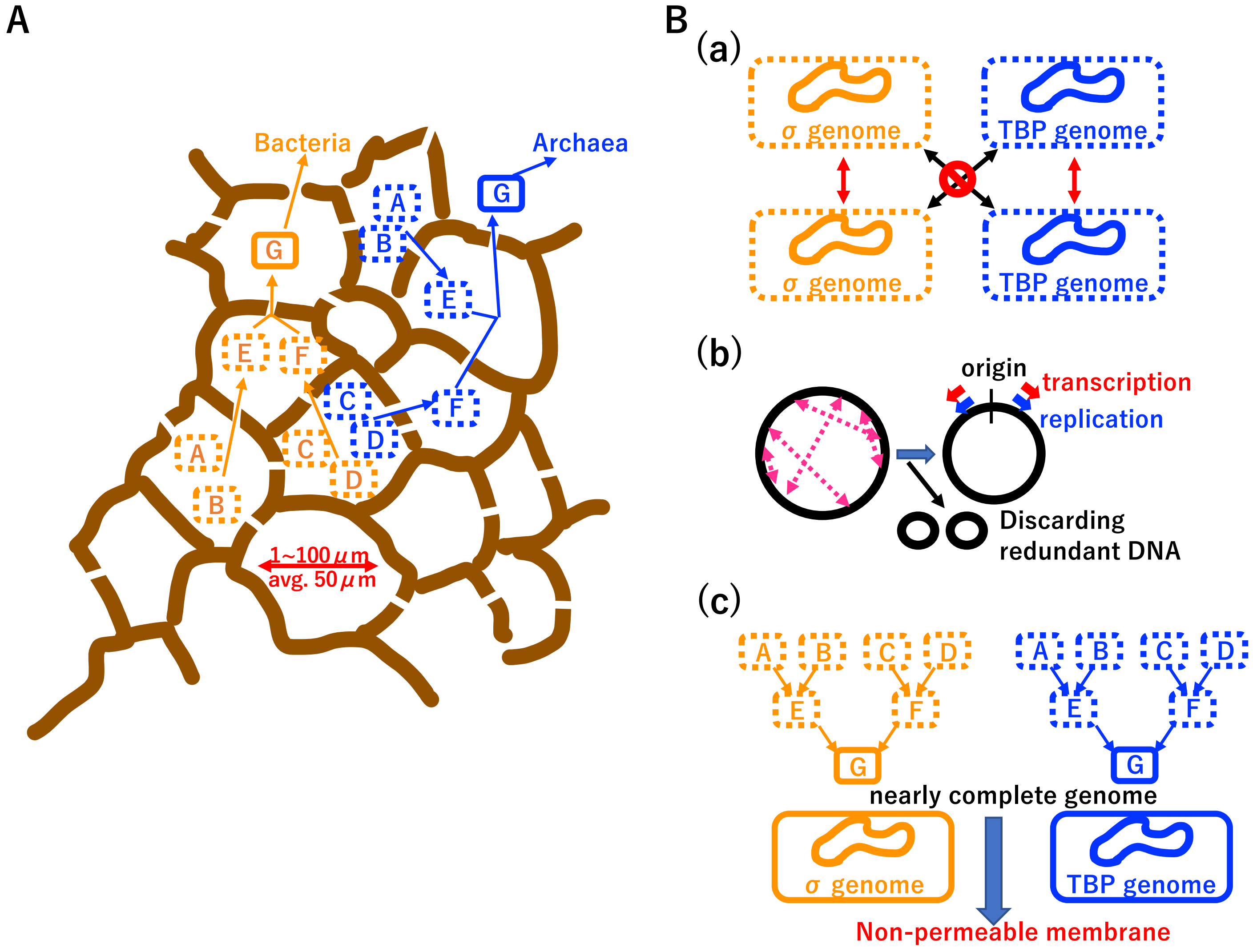

Supplementary Figure S13

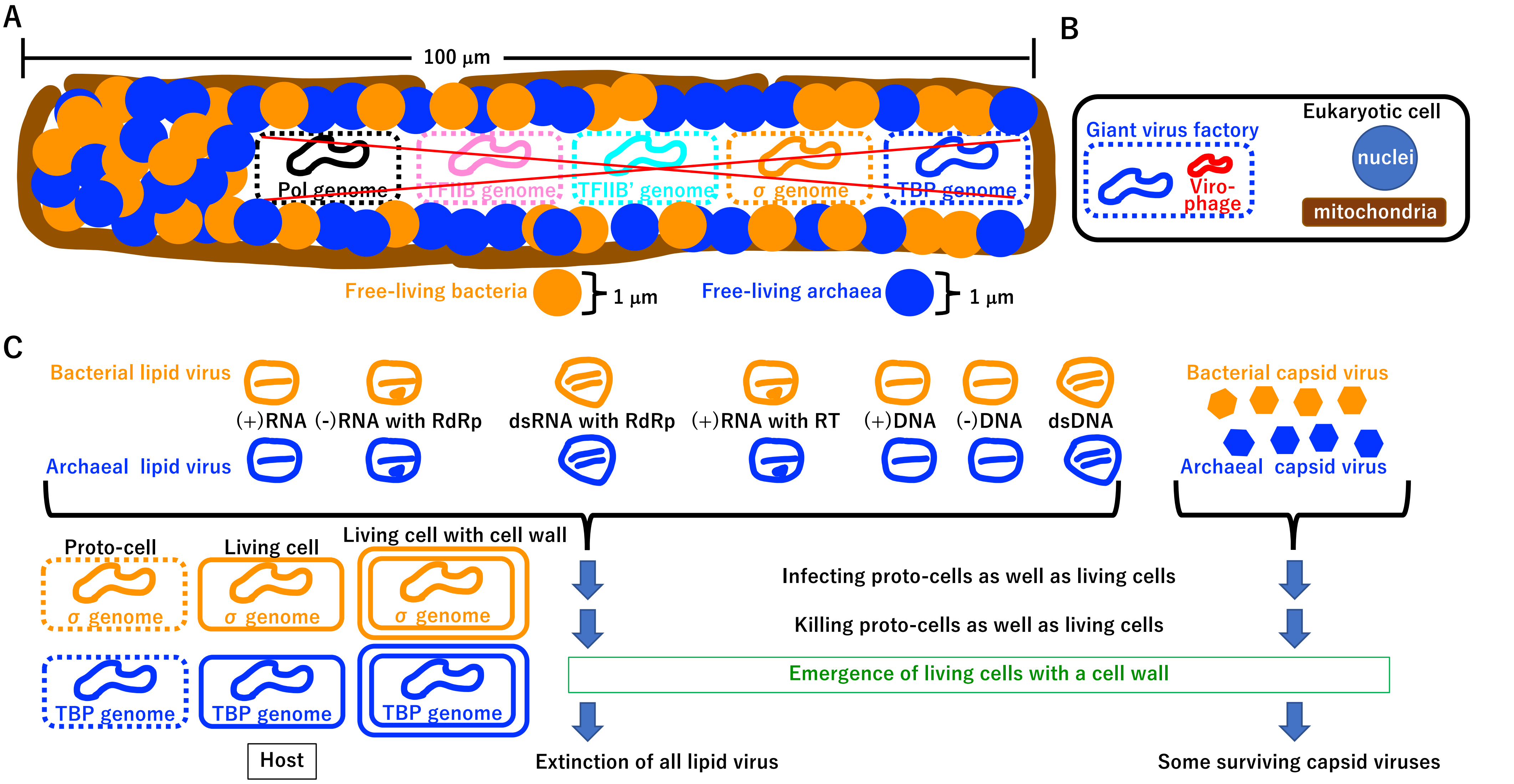

Supplementary Figure S14

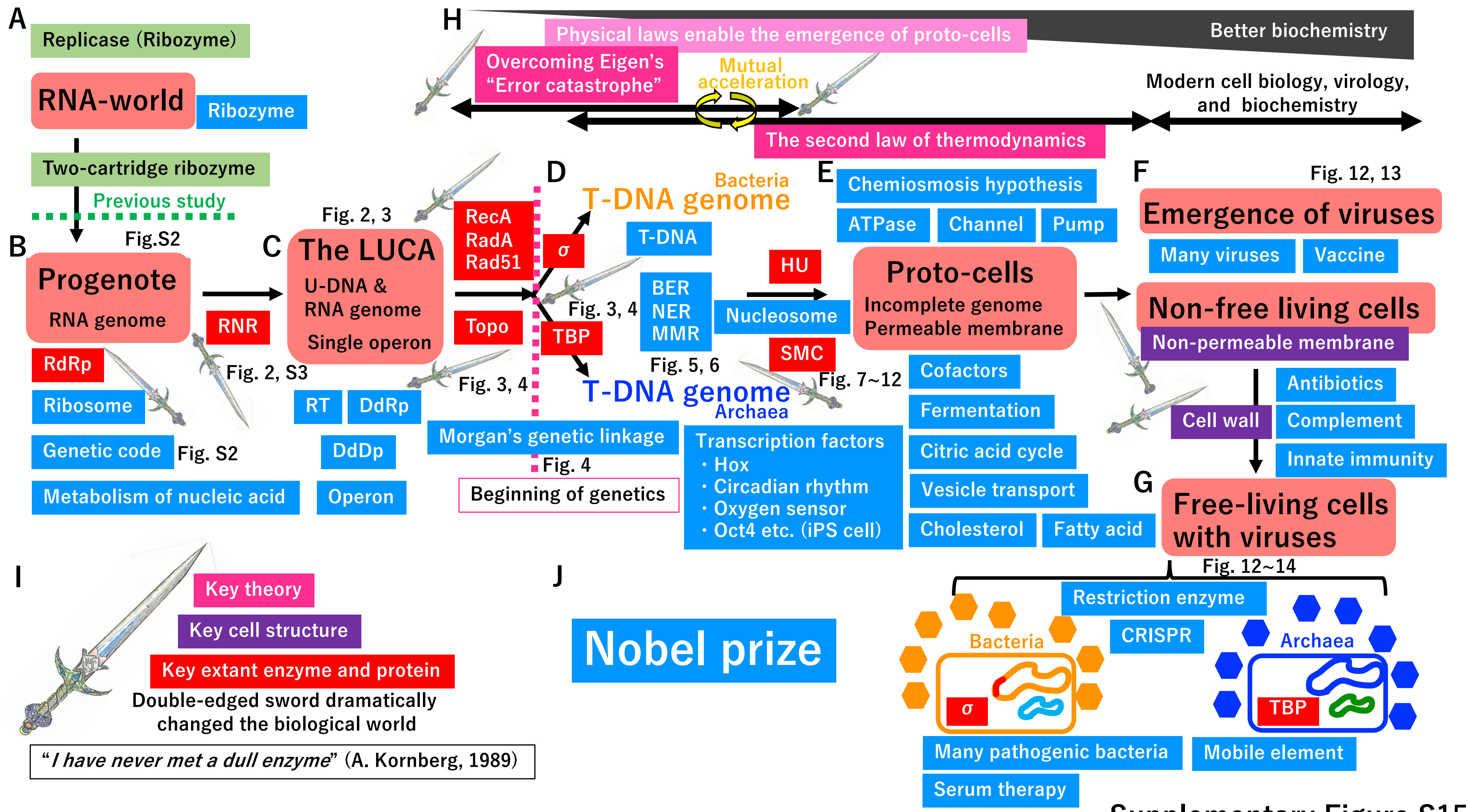

Supplementary Figure S15

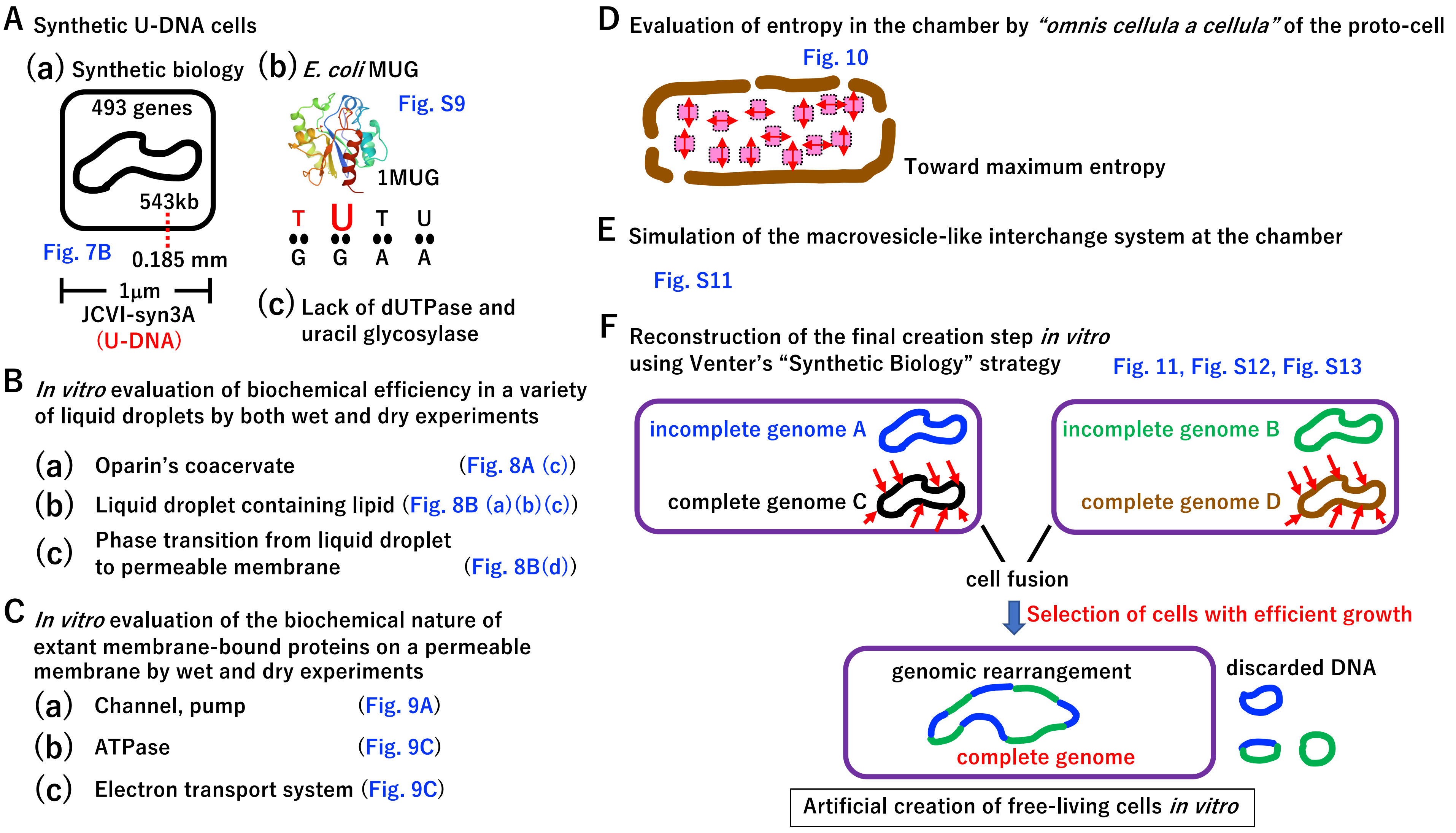

Supplementary Figure S16

(2) Supplementary figure legends

Supplementary Figure S1. Submarine alkaline hydrothermal vent

(A) Cartoon of a submarine alkaline hydrothermal vent (AHV). A submarine AHV was proposed as a candidate for the birth place of life [1, 2].

(B) Possible prebiotic reactions in an AHV. (a) Protons from a Hadean ocean. (b) Electrons derived from alkaline hypothermal fluid. (c) Green rust [3, 4]. Green rust forms double layer oxyhydroxide (DLH). (d)(e) Both protons and electrons drive prebiotic chemical reactions (from NO_3^- to $[CH_3H_6O_2NH_2]_n$) on DLH of green rust. (f) A reactor mimicking the conditions in an AHV produces compounds including ribose and deoxyribose [5]. (g) DLH made from $Mg_3Al(OH)_8$ facilitates production of peptides up to 24mer long *in vitro* [6].

Supplementary Figure S2. Progenote

(A) Genetic code and translation system. Alongside a variety of theories about the origin of the genetic code including those of Harrison [7], Yarus [8], and Lei & Burton [9-11], we proposed the concreate and reasonable theory [12]. Bacteria and Archaea share a genetic code and translation system. There are 34 ribosomal proteins shared by both lineages.

(B) Single operon-type mRNA. The common ribosomal protein S10 is encoded by the S10 operon in both lineages [13], suggesting that a single operon-type mRNA existed in the common ancestor of both lineages. There were around 500 genes in the LUCA [14, 15]. The latest estimate of the number of genes in the LUCA is 2,600 [16], but we use a smaller estimate in this study.

(C) Maximum length of single operon-type mRNA. Extant SARS-CoV-2 has a 30 kb RNA genome [17], one of largest genomes of RNA viruses. Thus, it is likely that 30 kb was the maximum size of single operon-type RNA in the ancient world.

(D) A chamber of the submarine alkaline hydrothermal vent (AHV). The progenote [18] could be surrounded by an inorganic chamber, with a similar size to that of extant eukaryotic cells (see Figure 1A). An RNA world, which could be a progenitor of the progenote, has been repeatedly denied [19-22]. However, the progenote as well as an RNA world (including a peptide-RNA world [23, 24]) might have somehow survived in an AHV because an AHV could continuously supply protons, electrons, metals, and a variety of prebiotic resources (including ribose/deoxyribose and peptides) (Supplementary Figure 1B). With an endless supply of energy and resources in an AHV, the progenote might have evolved to contain the extant metabolic map (except DNA and lipid metabolism), supported by enzymes encoded by the pool of RNA.

Supplementary Figure S3. Darwinian selection on the transition from RNA to U-DNA.

(A) Structure of RNR. Structure of *E. coli* ribonucleotide reductase (RNR) is presented with the PDB number. It is possible that there were no opportunities for the spontaneous emergence of sophisticated and complex enzymes, like RNR.

(B) Prebiotic synthesis of deoxyribonucleotides. Since the conversion from ribonucleotides to deoxyribonucleotides has been demonstrated *in vitro* in a prebiotic synthesis manner [25-28], the small number of dNTPs could trigger coevolution among (C), (D), (E), and (A), as described in the text.

(C) (D) (E) are the same as in Figure 2.

Supplementary Figure S4. Variety of replicases. As described previously [29-32], various replicases in the three domains of life are summarized. YES, indicates existence. n.d. indicates not described in the above references. Orange background indicates bacterial lineages. By contrast, a blue background indicates archaeal lineages.

Supplementary Figure S5. Establishment of homologous recombination.

(A) Single-stranded and double-stranded breaks. (a) Origin of DNA ligases. DNA polymerase at SSB (single-stranded break) on the template leads to lethal damage, double stranded-break (DSB). To prevent such DSB and complete DNA replication, two types of DNA ligases could simultaneously arise [33]. (b) DSB. If DSBs occur in dsDNA, the broken DNA would be inert. Live dsDNA is marked 1 and inert dsDNA is marked 0. (c) The degradation of abnormal macromolecules is beneficial for metabolism due to the reuse of monomer molecules. Thus, nuclease-mediated broken DNA degradation could emerge in the ancient world. Since Mre11/Rad50-type nucleases commonly exist in Bacteria and Archaea [34, 35], they might be among the nucleases that arose in these taxa. (d) Birth of recombinase. Degradation intermediates of dsDNA might provide a basis for the evolution of recombinase, an ancestor of recA, radA, or Rad51 in Bacteria, Archaea, or Eucarya, respectively [36, 37]. Of note, the synapsis of two dsDNA inactivates the template (marked by 0) for DNA replication and transcription.

(B) Completion of HR. (a) Synapsis, DNA synthesis, and ligation. Intermediate A(d), after synapsis, could be a template for DNA polymerase (Figure 2, Supplementary Figure S4) and subsequent DNA ligation (A)(a), leading to a double Holliday junction-type intermediate. Importantly, such an intermediate might exhibit a lack of activity (marked 0). (b) Recombinases itself, such as recA and Rad51, have primitive branch migration activity [38, 39]. Since evolution is not linear, dHJ (inert intermediate marked 0) might be degraded and reused for mono deoxynucleotides. Particular digestion of dHJ could yield two active dsDNAs (marked 2). (c) Robustness in the LUCA. Importantly, dsDNA produced the same RNA in the LUCA (Figure 3C). Thus, null intermediates (A)(d), (B)(a), and (B)(b) might equally survive in the LUCA. Such a robust system could support the evolution of a

complicated series of homologous recombination reactions. Branch migration and dHJ resolution by RuvABC [40] could have arisen specifically in bacterial lineages.

(C) Basic tool kit for the completion of dsDNA replication on damaged DNA. Transletion polymerase PolY (Figures 2J, 2K, Supplementary Figure S4) could mediate replication on damaged templates [41]. SSB on the template could be sealed by DNA ligase before encountering DNA polymerase. DSBs on dsDNA could be repaired by using homologous dsDNA in the chamber of the vent. Thus, the primitive tool kit for the completion of dsDNA replication could be established under the robust circumstances of the LUCA.

Supplementary Figure S6. Recombination-mediated genome rearrangement.

(A) Operon fusion. Crossing over-mediated homologous recombination facilitates the fusion of two operons.

(B) Gene fusion. Upon HR-mediated fusion between gene A and gene B, a novel fusion protein could easily emerge.

(C) Internal duplication. HR-mediated internal duplication could create dnaB from recA (Figure 5A) [42].

(D) Replication slippage-mediated internal duplication. TFIIB and TBP have internal duplications (Figure 3A) [43]. Since we predict that such duplications occurred before the establishment of HR, they might have occurred by replication slippage [44].

(E) Gene duplication. Ohno originally proposed that gene duplication facilitates evolution [45]. (a) Original gene. (b) Duplicated gene. (c) Gene maintaining the original function. (d) Gene with a gain-of-function mutation.

(F) Genome rearrangement. (a) Insertion and deletion mediated by HR. (b) Inversion by HR. (c) New genome. Two genomes (orange and blue) could be fused and undergo rearrangements and gene loss, yielding a new genome. Whole genome rearrangement requires HR.

Supplementary Figure S7. Transcriptional regulation.

(A) Copy number of nucleic acids regulates the amount of protein. (a) Seven types of nucleic acids might directly or indirectly produce mRNA, which is translated into protein in the LUCA (Figure 3C). (b) (c) Amounts of protein A and B. The amount of protein A could be ten-fold larger than that of protein B due to the amount of mRNA derived from operons A and B.

(B) Two operon fusion. After the fusion of dsDNA on operons A and B, one copy of DNA should produce a ten-fold difference between levels of protein A and B. Transcriptional regulation mediated by transcription factors [46] could emerge immediately after this fusion event. The basic unit of transcriptional regulation is promoter recognition (Pol, σ, TFIIB, TFIIB', and TBP/TFIIB') (Figure 3A). Since the same promoter recognition system

might be beneficial for the development of transcriptional regulatory networks (B), the transcriptional regulation divide should be accelerated (C) (Figure 3A).

(C) Transcriptional divide based on the promoter recognition system.

(D) σ genome and TBP genome. The σ [47] and TBP [48] genomes could give rise to Bacteria and Archaea, respectively. Bacterial proteins cannot be exchanged with archaeal proteins. Such incompatibility between lineages might have begun at the time of the two-operon fusion (B) (Figures 4B, 4C, Figure S6A).

Supplementary Figure S8. Establishment of Jacob's replicon.

(A) Ideal origin defined by genome rearrangements on circular dsDNA. (a) To avoid the terminal replication problem, a circular dsDNA genome could emerge on both the σ and TBP genome. (b) Bi-directional replication. Compared to unidirectional replication, bi-directional replication could speed up the completion of DNA replication of the whole genome. To minimize collision between replication and transcription (Figure 5A(b)), the direction of replication and transcription tends to be the same [49] via HR. (c) Two system fusion. Genomes carrying the same transcriptional operation system could be preferentially fused. (d) Novel fused genome. The fused genome could undergo rearrangement by HR (left column) as well as the loss of redundant genes. A new genome (right column) could arise under the same process (b).

(B) Evolution of an origin firing mechanism. (a) Mutation at the origin. A pre-defined origin on a new genome (A(d)) could acquire mutations toward an AT-rich sequence for easy opening. (b) Firing by RNA polymerase. The origin could be opened by RNA polymerase, similar to extant ColE1 plasmid replication [50]. (c) Firing by recombination. The origin could be opened by recombination, similar to T4 phage replication [51]. (d) Evolution of initiators. dnaA [52] and Orc/Cdc6 [53] could arise independently in the σ and TBP genomes, respectively. The initial initiator might only bind the replication origin. Later, interactions between the initiator and replicative DNA helicase could evolve [54-59]. Thus, the replicon model for DNA replication [60-62] could be established in a step-by-step manner, as described in Figures 2, 5, and Supplementary Figure S8. An extreme case of an initiator-helicase interaction is achieved in SV40 and polyoma virus T-antigen [63, 64].

(C) Unlinking of duplicated DNA. After the completion of DNA replication on the circular DNA genome, two daughter DNAs are topologically linked. The unlinking of daughter DNA by C/D and Xer A might emerge in Bacteria and Archaea, respectively [65].

Supplementary Figure S9. Possible scenario for the transition from U-DNA to T-DNA.

(A) Spontaneous deamination. 5-Methylcytosine and cytosine can be spontaneously deaminated, yielding thymine and uracil, respectively [66].

(B) Evolution of mismatch-specific uracil glycosylase (MUG). (a) *E. coli* MUG (structure shown with PDB ID) recognizes U/G and T/G mismatches. (b) *D. radiodurans* MUG (structure shown with PDB ID) recognizes the U/G mismatch and U/A non-mismatch [67]. (c) Possible substrate specificity of ancient MUG. We hypothesize that ancient MUG commonly emerged in both the σ and TBP genomes and has similar substrate specificity to that of *D. radiodurans* MUG. The U/T non-mismatch pair can be cut by the postulated ancient MUG. If the hypothesis is correct, there are three scenarios for evolution in the ancient alkaline vent. First, discarding the toxic *D. radiodurans* MUG-type enzyme led to cytosine deamination in the U-DNA genome, resulting in extinction. Second, keeping the *D. radiodurans* MUG-type enzyme drove the U-DNA to T-DNA transition (C–E). Third, changing the substrate specificity of *D. radiodurans* MUG toward that of *E. coli* MUG resolved the cytosine damination problem in the U-DNA genome. Although the third scenario predicts the existence of the U-DNA genome, cells with a U-DNA genome do not exist at present, suggesting that it went extinct. Since both extant bacteria (σ-genome) and archaea (TBP-genome) have T-DNA genomes, the second evolutionary route was most likely at the original alkaline vent.

(C) DNA polymerases efficiently utilize dUTP. DNA polymerases, including *E. coli* DNA polymerase I (Pol I), cannot discriminate between dTTP and dUTP as substrates for DNA synthesis [66], suggesting that the transition from U-DNA to T-DNA occurred without changing the DNA polymerase substrate specificity. The lack of time to change strongly suggests that rapid evolution increasing dTTP coupled with decreasing dUTP occurred in the ancient alkaline vent.

(D) Simultaneous evolution of TS and dUTPase. Metabolic map for dTTP synthesis is shown. Thymidylate synthetase (TS) might have evolved independently in the σ and TBP genome (F), leading to increased dTTP concentrations. By contrast, dUTPase decreases the dUTP concentration and supplies dUMP as a substrate for TS. The evolution of both TS and dUTPase enabled the transition from U-DNA to T-DNA.

(E) Elimination of mis-incorporated dUMP from the T-DNA genome. Due to the substrate specificity of DNA polymerase (C), dUMP can be frequently mis-incorporated into T-DNA. To eliminate uracil from T-DNA, mismatch-independent uracil glycosylase could arise. Coordinated activity of TS, dUTPase, and mismatch-independent uracil glycosylase-mediated BER could establish the T-DNA genome, whose structure was solved in 1953 [68].

(F) Possible steps in the transition from U-DNA to T-DNA. Summary of postulated events (B–E). After establishment of the T-DNA genome, the substrate specificities of *E. coli* and *D. radiodurans* MUGs were compatible with the T-DNA genome. Moreover, thymine DNA glycosylase might arise later.

Supplementary Figure S10. Electron transport system.

(A) Possible collection of outward proton transporters. External protons supplied from the inorganic chamber could trigger the evolution of inward and outward proton transport mechanisms [69, 70] based on the maximization of entropy inside and outside of proto-cells. (a)(b)(c) represent possible outward proton transporters. Importantly, any of these might equally contribute to proton transport.

(B) A variety of electron transport systems. In extant alkaline vents, electrons convert O_2 to H_2O. Under a lack of O_2 in ancient oceans, electrons could be transferred to surrounding metals at the vent (A). (a)(b) Electron transport on the permeable membrane of proto-cells without coupling to outward proton transport. Outward proton transport might be independently derived from electron transport. (c)–(e) A variety of electron transport systems coupled with outward proton transport. Importantly, any of these systems (a)–(e) might equally contribute to electron transport. System (e) could be selected in ancient cells (Figure 12B) because electron transport could be driven independently from the surrounding inorganic vent. Thus, independence from the original alkaline vent could be achieved by free-living cells.

Supplementary Figure S11. Postulated ancient vesicle transport system.

(A) Extant exosome and microvesicle in Eucarya. Membrane-based transport systems are present in Eucarya [71, 72].

(B) mRNA vaccines. An mRNA vaccine for the SARS-CoV-2 spike protein mimics the membrane-based transport system (A) [73].

(C) Extant bacteria/archaea extracellular exchange system. A variety of extra-membrane vesicles are exchanged among cells [74, 75]. In a biofilm, a nanotube containing multiple extra-membrane vesicles is connected between two different cells. EV: Extracellular vesicle, OMV: Outer membrane vesicle, O-IMV: Outer-inner membrane vesicle.

(D) The ESCRT system for vesicle-based transport. Some Archaea, such as Lokiarchaeota MK-D1, have the ESCRT system, suggesting that MK-D1 cells have an internal vesicle transport system, as in extant Eucarya. Some Archaea produce extramembrane vesicles (EVs) via the ESCRT system [75]. Moreover, enveloped archaeal viruses might bud from host cells via the ESCRT system [76]. It was proposed that ancient Archaea, like MK-D1 cells, undergo symbiosis with α-proteobacteria, leading to eucaryotic lineage cells [77].

(E) Postulated ancient vesicle transport system. Accounting for (A), (C), (D), and the concepts outlined in Figure 7B, a primitive macrovesicle-like transport system at the chamber of the vent was essential for survival of proto-cells. We hypothesize that ESCRT-dependent vesicle transport was invented at the ancient chamber. The postulated vesicle might carry any macromolecules.

Supplementary Figure S12. Puncted equilibrium at the cellular level.

(A) Evidence of system fusion and exchange during the history of life on Earth. (a) Three revolutionary system fusions. (1) Photosynthesis expending CO_2 and producing O_2 might be derived from the fusion of photosystems I and II. Event (1) created Cyanobacteria and irreversibly changed the conditions on Earth toward an O_2-available world. (2) Symbiosis between α-Proteobacteria and Archaea [78], like MK-D1 cells [77], led to the ancestor of extant Eucarya. (3) Symbiosis between Cyanobacteria and Eucarya led to an archaeplasrtid ancestor of extant plants [78]. (b) Giant virus-mediated system exchange. Extant giant virus carries a large DNA genome (up to 1,259 kbp) [79]. During symbiosis (2), the bacteriophage Tectiviridae (15 kb), whose host is α-Proteobacteria, might be introduced into the ancestor of Eucarya [80]. The coevolution of the ancestor of Eucarya and giant viruses could yield the LECA (last eukaryotic common ancestor). Eucarya has three DNA-dependent RNA polymerases (DdRp), I, II, and III. A phylogenetic analysis of DdRp revealed that multiple gene transfer events and rearrangements [81] created three DdRp(s) leading to the establishment of the LECA.

(B) Coevolution of the nucleosome structure. Just like DdRp, the nucleosome structure might also co-evolve in the ancestor of Eucarya and giant viruses. Representative nucleosome structures of Archaea [82], Eucarya, and giant viruses [83], presented with their PDB IDs.

Although Mimiviridae has a 1,259 kbp DNA genome, which is larger than the genome of JCVI-syn3, the minimum cell, giant viruses including Mimivoridae never reached free-living cells over 2 billion years. By contrast, free-living Bacteria and free-living Archaea emerged simultaneously in a narrow time window (an estimated 200 million years). Thus, we hypothesize that system fusion consistent with punctuated equilibrium occurred in the ancient alkaline vent (Figure 11, Supplementary Figure S13).

Supplementary Figure S13. Emergence of a nearly complete genome with a non-permeable membrane.

(A) Cascade of proto-cell fusion. Proto-cell E, the fusion product of proto-cells A and B, was fused to proto-cell F, the fusion product of proto-cell C and D, yielding cell G, with a nearly complete genome surrounded by a non-permeable membrane. The proto-cell and cell are surrounded by dashed (permeable membrane) and open (non-permeable membrane) boxes, respectively. Orange and blue cells represent bacterial and archaeal lineages, respectively.

(B) Crystallization process by a cascade of proto-cell fusion coupled with genome rearrangement. (a) Homologous system fusion. The same mechanisms underlying transcriptional regulation are preferentially fused (Figure 4). (b) Genome rearrangement. Upon the fusion of two systems, genome rearrangement occurs, as described in Supplementary Figure S8A. (c) Emergence of cells. After gaining a nearly complete

genome, a permeable membrane (dashed box) changed into a non-permeable membrane (solid box).

Supplementary Figure S14. Cell wall formation and the extinction of enveloped viruses.
Orange and blue represent bacterial and archaeal lineages, respectively.

(A) Free-living cells occupied all chambers of the original vent. All replicative intermediates described in Figures 2–11, including proto-cells, suffered from shortages of energy and resources. Most intermediates went extinct, except for some primitive viruses (Figure 13B).
(B) Virophage. Since extant virophages can infect giant viruses [84], primitive viruses in ancient vents also infected proto-cells as well as free-living cells.
(C) Cell wall formation. Among primitive viruses, enveloped viruses might have went extinct as a result of the inability to penetrate the cell wall. Some capsid viruses remained.

Supplementary Figure S15. New proposal for the crystallization process in the origin of bacteria, archaea, viruses, and mobile elements.
Main and supplementary figures related to each process involved in crystallization are marked in (B)–(H).

(A) RNA world. We proposed a mechanism for the change from an RNA world to a progenote (Supplementary Figure S2) [12]. A replicase made by RNA might arise [85-87]. A two-cartridge ribozyme might be a key for the evolution of the genetic code and translation system [12].
(B) Progenote. A key innovation for the maintenance of the progenote is RNA-dependent RNA polymerase (RdRp; PolB) (Supplementary Figure S2).
(C) The LUCA. The transition from the progenote to the LUCA required multiple enzymes, including ribonucleotide reductase (RNR) [88] (Figure 2A, Supplementary Figure S3). Enlargement of U-DNA might require a recombinase (Figure 3D) and topoisomerase (Figure 5A).
(D) Origin of genetics. Transcriptional initiation relied on σ or TBP in extant bacteria and archaea, respectively (Figure 3A). This divide begins at the initial stage of operon fusion, leading to genetic linkage (described by Morgan) and vertical inheritance (described by Mendel). Although the DNA replication machinery and DNA repair arose independently and simultaneously in both lineages (Figures 2, 5, 6), genes encoding common enzymes might have arisen in either lineage, followed by horizontal gene transfer. Since the size of the chamber in the original vent might be similar to the size of extant eukaryotic cells, enlarged DNA could be compacted by HU and histones in bacteria and archaea, respectively (Figure 6D). Further compaction could require SMC in both lineages (Figure 7D).

(E) Proto-cell. Compacted DNA might trigger coacervate [89] formation, lipid innovation, and proto-cells with a permeable membrane (Figure 8).

(F) Non-free-living cells with a nearly complete genome surrounded by a non-permeable membrane. Cells started to occupy all chambers in the vent in a selfish manner, leading to the emergence of primitive enveloped and capsid viruses. An arms race between non-free living cells and primitive viruses similar to that between extant cells and viruses might have occurred (Figures 12, 13).

(G) Free-living cells with viruses. Cell wall formation and independence from the inorganic vent yielded free-living cells with capsid viruses and mobile elements. During the course of cell wall formation, primitive enveloped viruses might have gone extinct (Figures 12, 13).

(H) Better biochemistry. The crystallization process could be driven by the laws of physics. The enlargement of DNA could overcome the "error-catastrophe" [90]. Proto-cell formation could be driven by the second law of thermodynamics. Overcoming "error-catastrophe" and the second law of thermodynamics could improve biochemical reactions and the crystallization process described in (D)–(F). Thus, σ and TBP genomes produce distinct lineages of free-living cells from the LUCA. Free-living cells occupied all chambers of the original vent. All replicative intermediates described in Figures 2–11, including proto-cells, suffered from a shortage of energy and resources. Most intermediates went extinct, except for some primitive viruses (Figure 13B).

(I) Key enzymes and proteins. Key theories, cell structures, and enzymes/proteins are marked by closed rectangles with a variety of colors. Kornberg stated "*I have never met a dull enzyme*" [91]. Enzymes that arose during the crystallization process in the original vent contributed to various intermediates, including free-living cells. Each innovation dramatically changed the destinies of developing life, symbolically marked as a double-edged sword.

(J) Not awarded a "Nobel prize". Advances in blue boxes have been awarded Nobel prizes, whereas those shown in red and pink boxes, which are keys for the emergence of free-living cells, have not. Forterre stated that "*the discovery of these enzymes (topoisomerases) was a great leap forward in our understanding of cell biology which may have deserved a Nobel Prize*" [92]. We believe that the key findings in the red and pink boxes, including the evolution of topoisomerases, are critical achievements.

Supplementary Figure S16. Possible *in vitro* and *in silico* experiments to test the hypotheses presented in this study. Corresponding figure numbers are marked in blue.

(A) Establish synthetic cells with U-DNA. Since *E. coli* MUG (Figure S9) can repair both T/G and U/G mismatches caused by the deamination of 5-methylcytosine and cytosine, deleting

genes encoding TS, dUTPase, or uracil glycosylase could generate cells carrying U-DNA by a synthetic biology approach (Figure 7B).

(B) Liquid droplets nucleated around compacted DNA. *In vitro* tests are need to evaluate whether compacted DNA can nucleate and form a coacervate and whether such a coacervate could recruit lipid components and yield a proto-cell-like structure surrounded by a permeable membrane. Many experiments using droplets [93-95] provide a basis for these analyses.

(C) Physical nature of channels, pumps, and ATPases on a permeable membrane. *In vitro* reconstruction of proto-cells is possible using vesicles with a permeable membrane, extant membrane bound proteins, and endless supplies of electrons, protons, and resources. We predict that extant membrane-bound proteins function efficiently under such conditions.

(D) Toward maximum entropy. Whether the principal of "*omnis cellula a cellula*" applies to proto-cells in the chamber of the alkaline vent under the second law of thermodynamics is an important question and can be evaluated by *in silico* experiments.

(E) Postulated vesicle transport system in the ancient vent. Although a variety of membrane-bound proteins evolved in the ancient vent, the transport of essential macromolecules inside and outside of proto-cells might be difficult. Thus, we hypothesized that a vesicle transport system was established in the ancient vent (Supplementary Figure S11). This hypothesis can be evaluated by *in silico* experiments.

(F) Reconstruction of the postulated final crystallization process. Using advanced synthetic biology approaches [96, 97], artificial fusion among proto-cells containing an incomplete genome could yield free-living cells. Artificially constructed incomplete genomes A and B can be introduced into each host cell. C and D, which are genomes of host cells, should be degraded during synthetic biology approaches.

(3) Supplementary references

1. Russell, M.J.; Hall, A.J.; Cairns-Smith, A.G.; Braterman, P.S. Sub-marine hot springs and the origin of life. *Nature* **1988**, *226*, 117.

2. Russell, M.J.; Hall, A.J. The emergence of life from iron monosulphide bubbles at a submarine hydrothermal redox and pH front. *J. Geol. Soc.* **1997**, *154*, 377-402.

3. Russell, M.J. Green rust: the simple organizing 'seed'of all life? *Life* **2018**, *8*, 35.

4. Duval, S.; Baymann, F.; Schoepp-Cothenet, B.; Trolard, F.; Bourrié, G.; Grauby, O.; Branscomb, E.; Russell, M.J.; Nitschke, W. Fougerite: The not so simple progenitor of the first cells. *Interface Focus* **2019**, *9*, 20190063.

5. Herschy, B.; Whicher, A.; Camprubi, E.; Watson, C.; Dartnell, L.; Ward, J.; Evans, J.R.; Lane, N. An origin-of-life reactor to simulate alkaline hydrothermal vents. *J. Mol. Evol.* **2014**, *79*, 213-227.

6. Erastova, V.; Degiacomi, M.T.; G Fraser, D.; Greenwell, H.C. Mineral surface chemistry control for origin of prebiotic peptides. *Nature commun.* **2017**, *8*, 2033.

7. Harrison, S. A.; Palmeira, R. N.; Halpern, A.; Lane, N. A biophysical basis for the emergence of the genetic code in protocells. *Biochim. Biophys. Acta -Bioenergetics* **2022**, *1863*, 148597.

8. Yarus, M. Getting past the RNA world: the initial Darwinian ancestor. *Cold Spring Harb. Perspect Biol.* **2011**, *3*, a003590

9. Lei, L.; Burton, Z.F. Evolution of life on earth: tRNA, aminoacyl-tRNA synthetases and the genetic code. *Life (Basel)* **2020**, *10*, doi:10.3390/life10030021.

10. Lei, L.; Burton, Z.F. Evolution of the genetic code. *Transcription* **2021**, *12*, 28-53.

11. Lei, L.; Burton, Z.F. "Superwobbling" and tRNA-34 wobble and tRNA-37 anticodon loop modifications in evolution and devolution of the genetic code. *Life (Basel)* **2022**, *12*, doi:10.3390/life12020252.

12. Seki, M. On the origin of the genetic code. *Genes Genet. Syst.* **2023**, *98*, 9–24.

13. Scholzen, T.; Arndt, E. Organization and nucleotide sequence of ten ribosomal protein genes from the region equivalent to the spectinomycin operon in the archaebacterium *Halobacterium marismortui*. *Mol. Gen. Genet.* **1991**, *228*, 70–80.

14. Koonin, E.V. Comparative genomics, minimal gene-sets and the last universal common ancestor. *Nat. Rev. Microbiol.* **2003**, *1*, 127–136.

15. Tuller, T.; Birin, H.; Gophna, U.; Kupiec, M.; Ruppin, E. Reconstructing ancestral gene content by coevolution. *Genome Res.* **2010**, *20*, 122–132.

16. Moody, E.R.R.; Alvarez-Carretero, S.; Mahendrarajah, T.A.; Clark, J.W.; Betts, H.C.; Dombrowski, N.; Szantho, L.L.; Boyle, R.A.; Daines, S.; Chen, X., et al. The nature of the last universal common ancestor and its impact on the early Earth system. *Nat. Ecol. Evol.* **2024**, *8*, 1654-1666.

17. Kokic, G.; Hillen, H.S.; Tegunov, D.; Dienemann, C.; Seitz, F.; Schmitzova, J.; Farnung, L.; Siewert, A.; Höbartner, C.; Cramer, P. Mechanism of SARS-CoV-2 polymerase stalling by remdesivir. *Nat. Commun.* **2021**, *12*, 279.

18. Woese, C. The universal ancestor. *Proc. Natl. Acad. Sci. USA.* 1998, *95*, 6854–6859.

19. Yockey, H.P. Comments on "Let there be life"; Thermodynamic reflections on biogenesis and evolution" by Avshalom C. Elitzur. J. *Theor. Biol.* **1995**, *176*, 349-355.

20. Mills, G.C.; Kenyon, D. The RNA world: A critique. *Origins and Design* **1996**, *17*, 138.237.

21. Kurland, C.G. The RNA dreamtime: Modern cells feature proteins that might have supported a prebiotic polypeptide world but nothing indicates that RNA world ever was. *Bioessays* **2010**, *32*, 866-871.

22. Wills, P.R.; Carter, C.W. Insuperable problems of the genetic code initially emerging in an RNA world. *Biosystems* **2018**, *164*, 155-166.

23. Muller, F.; Escobar, L.; Xu, F.; Wegrzyn, E.; Nainyte, M.; Amatov, T.; Chan, C.Y.; Pichler, A.; Carell, T. A prebiotically plausible scenario of an RNA-peptide world. *Nature* **2022**, *605*, 279-284.

24. Lei, L.; Burton, Z.F. The 3 31 nucleotide minihelix tRNA evolution theorem and the origin of life. *Life (Basel)* **2023**, *13*, doi:10.3390/life13112224.

25. Powner, M.W.; Zheng, S.L.; Szostak, J.W. Multicomponent assembly of proposed DNA precursors in water. *J. Am. Chem. Soc.* **2012**, *134*, 13889–13895.

26. Dragičević, I.; Barić, D.; Kovačević, B.; Golding, B.T.; Smith, D.M. Non-enzymatic ribonucleotide reduction in the prebiotic context. *Chemistry* **2015**, *21*, 6132–6143.

27. Kim, S.C.; Zhou, L.; Zhang, W.; O'Flaherty, D.K.; Rondo-Brovetto, V.; Szostak, J.W. A model for the emergence of RNA from a prebiotically plausible mixture of ribonucleotides, arabinonucleotides, and 2'-deoxynucleotides. *J. Am. Chem. Soc.* **2020**, *142*, 2317–2326.

28. Xu, J.; Chmela, V.; Green, N.J.; Russell, D.A.; Janicki, M.J.; Góra, R.W.; Szabla, R.; Bond, A. D.; Sutherland, J.D. Selective prebiotic formation of RNA pyrimidine and DNA purine nucleosides. *Nature* **2020**, *582*, 60–66.

29. Loc'h, J.; Gerodimos, C.A.; Rosario, S.; Tekpinar, M.; Lieber, M.R.; Delarue, M. Structural evidence for an in trans base selection mechanism involving Loop1 in polymerase μ at an NHEJ double-strand break junction. *J. Biol. Chem.* **2019**, *294*, 10579–10595.

30. Acharya, N.; Khandagale, P.; Thakur, S.; Sahu, J. K.; Utkalaja, B.G. Quaternary structural diversity in eukaryotic DNA polymerases: monomeric to multimeric form. *Curr. Genet.* **2020**, *66*, 635–655.

31. Koonin, E.V.; Krupovic, M.; Ishino, S.; Ishino, Y. The replication machinery of LUCA: common origin of DNA replication and transcription. *BMC Biol.* **2020**, *18*, 61.

32. de Farias, S.T.; Furtado, A.N.M.; Dos Santos Junior, A.P.; José, M.V. Natural history of DNA-dependent DNA polymerases: Multiple pathways to the origins of DNA. *Viruses* **2023**, *15*, 749.

33. Pergolizzi, G.; Wagner, G.K.; Bowater, R.P. Biochemical and structural characterisation of DNA ligases from bacteria and archaea. *Biosci. Rep.* **2016**, *36*, art:e00391.

34. Symington, L.S. End resection at double-strand breaks: mechanism and regulation. *Cold Spring Harb. Perspect Biol.* **2014**, *6*, a016436.

35. Paull, T.T. 20 years of Mre11 biology: No end in sight. *Mol. Cell* **2018**, *71*, 419–427.

36. Stasiak, A.; Di Capua, E. The helicity of DNA in complexes with recA protein. *Nature* **1982**, *299*, 185–186.

37. Shinohara, A.; Ogawa, H.; Ogawa, T. Rad51 protein involved in repair and recombination in S. cerevisiae is a RecA-like protein. *Cell* **1992**, *69*, 457–470.

38. Cunningham, R.P.; DasGupta, C.; Shibata, T.; Radding, C.M. Homologous pairing in genetic recombination: recA protein makes joint molecules of gapped circular DNA and closed circular DNA. *Cell* **1980**, *20*, 223–235.

39. Murayama, Y.; Kurokawa, Y.; Mayanagi, K.; Iwasaki, H. Formation and branch migration of Holliday junctions mediated by eukaryotic recombinases. *Nature* **2008**, *451*, 1018–1021.

40. Ariyoshi, M.; Vassylyev, D.G.; Iwasaki, H.; Nakamura, H.; Shinagawa, H.; Morikawa, K. Atomic structure of the RuvC resolvase: a holliday junction-specific endonuclease from *E. coli*. *Cell* **1994**, *78*, 1063–1072.

41. Sale, J.E.; Lehmann, A.R.; Woodgate, R. Y-family DNA polymerases and their role in tolerance of cellular DNA damage. *Nat. Rev. Mol. Cell Biol.* **2012**, *13*, 141–152.

42. Leipe, D.D.; Aravind, L.; Grishin, N.V.; Koonin, E.V. The bacterial replicative helicase DnaB evolved from a RecA duplication. *Genome Res.* **2000**, *10*, 5–16.

43. Adachi, N.; Senda, T.; Horikoshi, M. Uncovering ancient transcription systems with a novel evolutionary indicator. *Sci. Rep.* **2016**, *6*, 27922.

44. Yang, G.; Zheng, R.Y.; Tan, Q.; Dong, C.J.; Jin, Z.S. Clinical characteristics and responses to chemotherapy and immune checkpoint inhibitor treatment for microsatellite instability gastric cancer. *Am. J. Cancer Res.* **2020**, *10*, 4123–4133.

45. Ohno, S.; Wolf, U.; Atkin, N.B. Evolution from fish to mammals by gene duplication. *Hereditas* **1968**, *59*, 169–187.

46. Jacob, F.; Monod, J. 1961. Genetic regulatory mechanisms in the synthesis of proteins. *J. Mol. Biol.* **1961**, *3*, 318–356.

47. Sugiura, M.; Okamoto, T.; Takanami, M. RNA polymerase sigma-factor and the selection of initiation site. *Nature* **1970**, *225*, 598–600.

48. Horikoshi, M.; Wang, C.K.; Fujii, H.; Cromlish, J.A.; Weil, P.A.; Roeder, R.G. Cloning and structure of a yeast gene encoding a general transcription initiation factor TFIID that binds to the TATA box. *Nature* **1989**, *341*, 299–303.

49. Blattner, F.R.; Plunkett III.G.; Bloch, C.A.; Perna, N.T.; Burland, V.; Riley, M.; Collado-Vides, J.; Glasner, J.D.; Rode, C.K.; Mayhew, G.F.; Gregor, J.; Davis, N.W.; Kirkpatrick, H.A.; Goeden, M.A.; Rose, D.J.; Mau, B.; Shao, Y. The complete genome sequence of *Escherichia coli* K-12. *Science* **1997**, *277*, 1453–1474.

50. Masukata, H.; Tomizawa, J. Control of primer formation for ColE1 plasmid replication: conformational change of the primer transcript. *Cell* **1986**, *44*, 125–136.

51. Formosa, T.; Alberts, B.M. DNA synthesis dependent on genetic recombination: characterization of a reaction catalyzed by purified bacteriophage T4 proteins. *Cell* **1986**, *47*, 793–806.

52. Fuller, R.S.; Kornberg, A. Purified dnaA protein in initiation of replication at the *Escherichia coli* chromosomal origin of replication. *Proc. Natl. Acad. Sci. USA.* **1983**, *80*, 5817–5821.

53. Bell, S.P.; Mitchell, J.; Leber, J.; Kobayashi, R.; Stillman, B.The multidomain structure of Orc1p reveals similarity to regulators of DNA replication and transcriptional silencing. *Cell* **1995**, *83*, 563–568.

54. Bramhill, D.; Kornberg, A. A model for initiation at origins of DNA replication. *Cell* **1987**, *54*, 915–918.

55. Blow, J.J.; Laskey, R.A. A role for the nuclear envelope in controlling DNA replication within the cell cycle. *Nature* **1988**, *332*, 546–548.

56. Kubota, Y.; Mimura, S.; Nishimoto, S.; Takisawa, H.; Nojima, H. Identification of the yeast MCM3-related protein as a component of *Xenopus* DNA replication licensing factor. *Cell* **1995**, *81*, 601–609.

57. Nishitani, H.; Lygerou, Z.; Nishimoto, T.; Nurse, P. The Cdt1 protein is required to license DNA for replication in fission yeast. *Nature* **2000**, *404*, 625–628.

58. Tada, S.; Li, A.; Maiorano, D.; Méchali, M.; Blow, J.J. Repression of origin assembly in metaphase depends on inhibition of RLF-B/Cdt1 by geminin. *Nat. Cell Biol.* **2001**, *3*, 107–113.

59. Hayashi, C.; Miyazaki, E.; Ozaki, S.; Abe, Y.; Katayama, T. DnaB helicase is recruited to the replication initiation complex via binding of DnaA domain I to the lateral surface of the DnaB N-terminal domain. *J. Biol. Chem.* **2020**, *295*, 11131–11143.

60. Jacob, F. On regulation of DNA replication in Bacteria. *Cold Spring Harb. Quant. Biol.* **1963**, *28*, 329.

61. Michel, B.; Bernander, R. Chromosome replication origins: do we really need them? *Bioessays* **2014**, *36*, 585–590.

62. Masai, H. Replicon hypothesis revisited. *Biochem. Biophys. Res. Commun.* **2022**, *633*, 77–80.

63. Dean, F.B.; Bullock, P.; Murakami, Y.; Wobbe, C.R.; Weissbach, L.; Hurwitz, J. Simian virus 40 (SV40) DNA replication: SV40 large T antigen unwinds DNA containing the SV40 origin of replication. *Proc. Natl. Acad. Sci. USA.* **1987**, *84*, 16–20.

64. Seki, M.; Enomoto, T.; Eki, T.; Miyajima, A.; Murakami, Y.; Hanaoka, F.; Ui, M. DNA helicase and nucleoside-5'-triphosphatase activities of polyoma virus large tumor antigen. *Biochemistry* **1990**, *29*, 1003–1009.

65. Cortez, D.; Quevillon-Cheruel, S.; Gribaldo, S.; Desnoues, N.; Sezonov, G.; Forterre, P.; Serre, M.C. Evidence for a Xer/dif system for chromosome resolution in archaea. *PLoS Genet.* **2010**, *6*, e1001166.

66. Kornberg, A.; Baker, T. DNA Replication, Second Edition. *Freeman, New York.* **1992**.

67. Moe, E.; Leiros, I.; Smalås, A.O.; McSweeney, S. The crystal structure of mismatch-specific uracil-DNA glycosylase (MUG) from *Deinococcus radiodurans* reveals a novel catalytic residue and broad substrate specificity. *J. Biol. Chem.* **2006**, *281*, 569–577.

68. Watson, J.D.; Crick, F.H.C. Molecular structure of nucleic acids; a structure for deoxyribose nucleic acid. *Nature* **1953**, *171*, 737–738.

69. Lane, N.; Allen, J.F.; Martin, W. How did LUCA make a living? Chemiosmosis in the origin of life. *Bioessays* **2010**, *32*, 271–280.

70. Martin, W.F.; Sousa, F.L.; Lane, N. Evolution. Energy at life's origin. *Science* **2014**, *344*, 1092–1093.

71. Tkach, M.; Théry, C. Communication by extracellular vesicles: Where we are and where we need to go. *Cell* **2016**, *164*, 1226–1232.

72. Mathieu, M.; Martin-Jaular, L.; Lavieu, G.; Théry, C. Specificities of secretion and uptake of exosomes and other extracellular vesicles for cell-to-cell communication. *Nature Cell Biol.* **2019**, *21*, 9–17.

73. Hoffmann, M.A.G.; Yang, Z.; Huey-Tubman, K.E.; Cohen, A.A.; Gnanapragasam, P.N.P.; Nakatomi, L.M.; Storm, K.N.; Moon, W.J.; Lin, P.J.C.; West Jr.A.P.; Bjorkman, P.J. ESCRT recruitment to SARS-CoV-2 spike induces virus-like particles that improve mRNA vaccines. *Cell* **2023**, *186*, 2283–2492.

74. Gill, S.; Catchpole, R.; Forterre, P. Extracellular membrane vesicles in the three domains of life and beyond. *FEMS Microbiol. Rev.* **2019**, *43*, 273–303.

75. Liu, J.; Cvirkaite-Krupovic, V.; Commere, P.H.; Yang, Y.; Zhou, F.; Forterre, P.; Shen, Y.; Krupovic, M. Archaeal extracellular vesicles are produced in an ESCRT-dependent manner and promote gene transfer and nutrient cycling in extreme environments. *ISME J.* **2021**, *15*, 2892–2905.

76. Quemin, E.R.; Chlanda, P.; Sachse, M.; Forterre, P.; Prangishvili, D.; Krupovic, M. Eukaryotic-like virus budding in archaea. *mBio.* **2016**, *7*, e01439-16.

77. Imachi, H.; Nobu, M.K.; Nakahara, N.; Morono, Y.; Ogawara, M.; Takaki, Y.; Takano, Y.; Uematsu, K.; Ikuta, T.; Ito, M.; Matsui, Y.; Miyazaki, M.; Murata, K.; Saito, Y.; Sakai, S.; Song, C.; Tasumi, E.; Yamanaka, Y.; Yamaguchi, T.; Kamagata, Y.; Tamaki, H.; Takai, K. Isolation of an archaeon at the prokaryote-eukaryote interface. *Nature* **2020**, *577*, 519–525.

78. Margulis, L. Symbiosis and evolution. *Sci. Am.* **1971**, *225*, 48–57.

79. Queiroz, V.F.; Rodrigues, R.A.L.; de Miranda Boratto, P.V.; La Scola, B.; Andreani, J.; Abrahão, J.S. Amoebae: Hiding in plain sight: Unappreciated hosts for the very large viruses. *Annu. Rev. Virol.* **2022**, *9*, 79–98.

80. Koonin, E.V.; Yutin, N. Evolution of the large nucleocytoplasmic DNA viruses of eukaryotes and convergent origins of viral gigantism. *Adv. Virus Res.* **2019**, *103*, 167–202.

81. Guglielmini, J.; Woo, A.C.; Krupovic, M.; Forterre, P.; Gaia, M. Diversification of giant and large eukaryotic dsDNA viruses predated the origin of modern eukaryotes. *Proc. Natl. Acad. Sci. USA.* **2019**, *116*, 19585–19592.

82. Mattiroli, F.; Bhattacharyya, S.; Dyer, P.N.; White, A.E.; Sandman, K.; Burkhart, B.W.; Byrne, K.R.; Lee, T.; Ahn, N.G.; Santangelo, T.J.; Reeve, J.N.; Luger, K. Structure of histone-based chromatin in Archaea. *Science* **2017**, *357*, 609–612.

83. Liu, Y.; Bisio, H.; Toner, C.M.; Jeudy, S.; Philippe, N.; Zhou, K.; Bowerman, S.; White, A.; Edwards, G.; Abergel, C.; Luger, K. Virus-encoded histone doublets are essential and form nucleosome-like structures. *Cell* **2021**, *184*, 4237–4250.

84. La Scola, B.; Desnues, C.; Pagnier, I.; Robert, C.; Barrassi, L.; Fournous, G.; Merchat, M.; Suzan-Monti, M.; Forterre, P.; Koonin, E.V.; Raoult, D. The virophage as a unique parasite of the giant mimivirus. *Nature* **2008**, *455*, 100–104.

85. Bartel, D.P.; Doudna, J.A.; Usman,N.; Szostak, J.W. Template-directed primer extension catalyzed by the *Tetrahymena* ribozyme. *Mol. Cell. Biol.* **1991**, *11*, 3390–3394.

86. Been, M.D.; Cech, T.R. RNA as an RNA polymerase: net elongation of an RNA primer catalyzed by the Tetrahymena ribozyme. *Science* **1988**, *239*, 1412–1416.

87. McGinness, K.E.; Joyce, G.F. In search of an RNA replicase ribozyme. *Chem. Biol.* **2003**, *10*, 5–14.

88. Greene, B.L.; Kang, G.; Cui, C.; Bennati, M.; Nocera, D.G.; Drennan, C.L.; Stubbe, J. Ribonucleotide reductases: Structure, chemistry, and metabolism suggest new therapeutic targets. *Annu. Rev. Biochem.* **2020**, *89*, 45–75.

89. Oparin, A.I. The origin of life. *McMillan, New York, USA.***1938**.

90. Eigen, M. Self-organization of matter and the evolution of biological macromolecules. *Naturwissenschaften* **1971**, *58*, 465–523.

91. Kornberg, A. Never a dull enzyme. *Annu. Rev. Biochem.* **1989**, *58*, 1–30.

92. Forterre, P.; Gribaldo, S.; Gadelle, D.; Serre, M.C. Origin and evolution of DNA topoisomerases. *Biochimie* **2007**, *89*, 427–446.

93. Courchaine, E.M.; Lu, A.; Neugebauer, K.M. Droplet organelles? *EMBO J.* **2016**, *35*, 1603–1612.

94. Feng, Z.Y.; Liu, T.T.; Sang, Z.T.; Lin, Z.S.; Su, X.; Sun, X. T.; Yang, H.Z.; Wang, T.; Guo, S. Microfluidic preparation of janus microparticles with temperature and pH triggered degradationproperties. *Front. Bioeng. Biotechnol.* **2021**, *9*. 756758.

95. Xu, C.; Martin, N.; Li, M.; Mann, S. Living material assembly of bacteriogenic protocells. *Nature* **2022**, *609*, 1029–1037.

96. Olivi, L.; Berger, M.; Creyghton, R.N.P.; De Franceschi, N.; Dekker, C.; Mulder, B.M.; Claassens, N.J.; Ten Wolde, P.R.; van der Oost, J. Towards a synthetic cell cycle. *Nat. Commun.* **2021**, *12*, 4531.

97. Venter, J.C.; Glass, J.I.; Hutchison, C.A.3rd.; Vashee, S. Synthetic chromosomes, genomes, viruses, and cells. *Cell* **2022**, *185*, 2708–2724.
